# Re-expression of *REG* family and *DUOX*s genes in CRC organoids by co-culturing with CAFs

**DOI:** 10.1038/s41598-021-81475-2

**Published:** 2021-01-22

**Authors:** Mie Naruse, Masako Ochiai, Shigeki Sekine, Hirokazu Taniguchi, Teruhiko Yoshida, Hitoshi Ichikawa, Hiromi Sakamoto, Takashi Kubo, Kenji Matsumoto, Atsushi Ochiai, Toshio Imai

**Affiliations:** 1 Central Animal Division, Fundamental Innovative Oncology Core, National Cancer Center Research Institute, 5-1-1 Tsukiji, Chuo-ku, Tokyo, 104-0045 Japan; 2 Department of Diagnostic Pathology, National Cancer Center Hospital, 5-1-1 Tsukiji, Chuo-ku, Tokyo, 104-0045 Japan; 3 Department of Clinical Genomics, Fundamental Innovative Oncology Core, National Cancer Center Research Institute, 5-1-1 Tsukiji, Chuo-ku, Tokyo, 104-0045 Japan; 4 Department of Allergy and Clinical Immunology, National Research Institute for Child Health and Development, 2-10-1 Okura, Setagaya-ku, Tokyo, 157-8535 Japan; 5 Exploratory Oncology Research & Clinical Trial Center, National Cancer Center, 6-5-1 Kashiwanoha, Kashiwa, Chiba 277-8577 Japan

**Keywords:** Biological techniques, Cancer, Molecular biology

## Abstract

Organoids derived from epithelial tumors have recently been utilized as a preclinical model in basic and translational studies. This model is considered to represent the original tumor in terms of 3D structure, genetic and cellular heterogeneity, but not tumor microenvironment. In this study, we established organoids and paired cancer-associated fibroblasts (CAFs) from surgical specimens of colorectal carcinomas (CRCs), and evaluated gene expression profiles in organoids with and without co-culture with CAFs to assess interactions between tumor cells and CAFs in tumor tissues. We found that the expression levels of several genes, which are highly expressed in original CRC tissues, were downregulated in organoids but re-expressed in organoids by co-culturing with CAFs. They comprised immune response- and external stimulus-related genes, e.g., *REG* family and dual oxidases (*DUOX*s), which are known to have malignant functions, leading tumor cells to proliferative and/or anti-apoptotic states and drug resistant phenotypes. In addition, the degree of differential induction of *REG1* and *DUOX2* in the co-culture system varied depending on CAFs from each CRC case. In conclusion, the co-culture system of CRC organoids with paired CAFs was able to partially reproduce the tumor microenvironment.

## Introduction

Two-dimensional (2D) cancer cell lines that can be easily and reproducibly maintained in vitro have been mainly used for cancer studies targeting the clarification of molecular mechanisms of carcinogenesis or evaluation of anticancer drugs. As most cancer cell lines were cultured under over-nourished conditions, differing from the in vivo environment, and repeated passages, the characteristics of original tumor tissues, such as expression of stem cell markers and differentiation marker genes, and proliferation, invasion, and drug metabolic abilities are altered in cancer cell lines^1–3^. The low probability of success of clinical trials is partly due to the use of cancer cell lines in preclinical evaluations of drug candidates^3–6^. To promote efficient drug discovery, organoid culture systems have been utilized recently^7,8^.

An organoid is a miniaturized and simplified organ produced in vitro using 3D culture systems that resembles a realistic micro-organ. They are derived from one or a few cells from a tissue, and demonstrate natural cell–cell communication and undergo self-organization in 3D culture^9,10^. As an ex vivo model, cancer tissue-derived organoids are considered suitable to analyze direct reactivities of carcinoma cells to growth factors/cytokines, miRNAs, or synthetic compounds; however, stroma cells, including fibroblasts, immune cells, and vascular cells, which interact with carcinoma cells in original cancer tissues, are not maintained using this culture technique. Under such circumstances, co-culture systems of primary carcinoma cells with native stroma cells, e.g., tissue-embedded immune cells, have been introduced^11^.

The interactions between cancer cells and cancer associated fibroblasts (CAFs) vary and are considered to be important for carcinogenesis^12–14^. Moreover, the epithelial mesenchymal transition (EMT), which enables tumor cells to acquire resistance to anti-cancer drugs, was reported to be facilitated by the presence of CAFs^15–17^. To date, there have been limited investigations using co-culture systems of 3D-cultured cancer cells with CAFs in several types of cancers, e.g., pancreatic ductal adenocarcinomas^18,19^, prostate adenocarcinomas^20^, and esophageal carcinomas^21^, and interactions between cancer cells and CAFs were demonstrated to affect cancer aggressiveness and resistance to anti-cancer drugs.

The purpose of the present study was to clarify if the co-culture system of CRC organoids with CAFs reproduces the microenvironment between tumor cells and CAFs observed in the original cancer tissues. First, comprehensive gene expression analyses between original tumor tissues and organoids were performed to characterize the features of the organoid culture system. Second, to identify gene expression changes in organoids induced by co-culturing with CAFs, paired CAFs from each case were established, and gene expression profiles between organoids with or without associated CAFs were compared. The present comparative screening of gene expression profiles of clinical tissue-derived CRC organoids with or without corresponding patient tissue-derived CAFs is highly important because the major molecules involved in interactions between tumor cells and CAFs have not been comprehensively evaluated using 3D-cultured CRC organoids and paired CAFs, which have been reported to vary among cases^22,23^.

## Results

### Baseline characteristics of CRC

Basic patient and pathological data in the 45 CRC cases used for establishment of organoids and fibroblasts are summarized in Supplementary Table S1. Of the 45 cancer patients, 60% were males and 40% were females. This male per female ratio is comparable with Japanese Cancer Statistics (Cancer Registry and Statistics. Cancer Information Service, National Cancer Center, Japan (Monitoring of Cancer Incidence in Japan (MCIJ)). Average ages were 62 years old, ranging from 36 years old to 88 years old. The proportions of stage I, stage II, stage III, and stage IV cancers were 7%, 47%, 31%, and 15%, respectively, and 85% were found in the left colon (D, S, Rs, Ra, and Rb) and 15% in the right colon (T, A, and C). The detailed baseline characteristics of donor patients are summarized in Supplementary Table S1 and Supplementary Fig. S1.

### Establishment of organoids and fibroblasts

**Figure 1:**
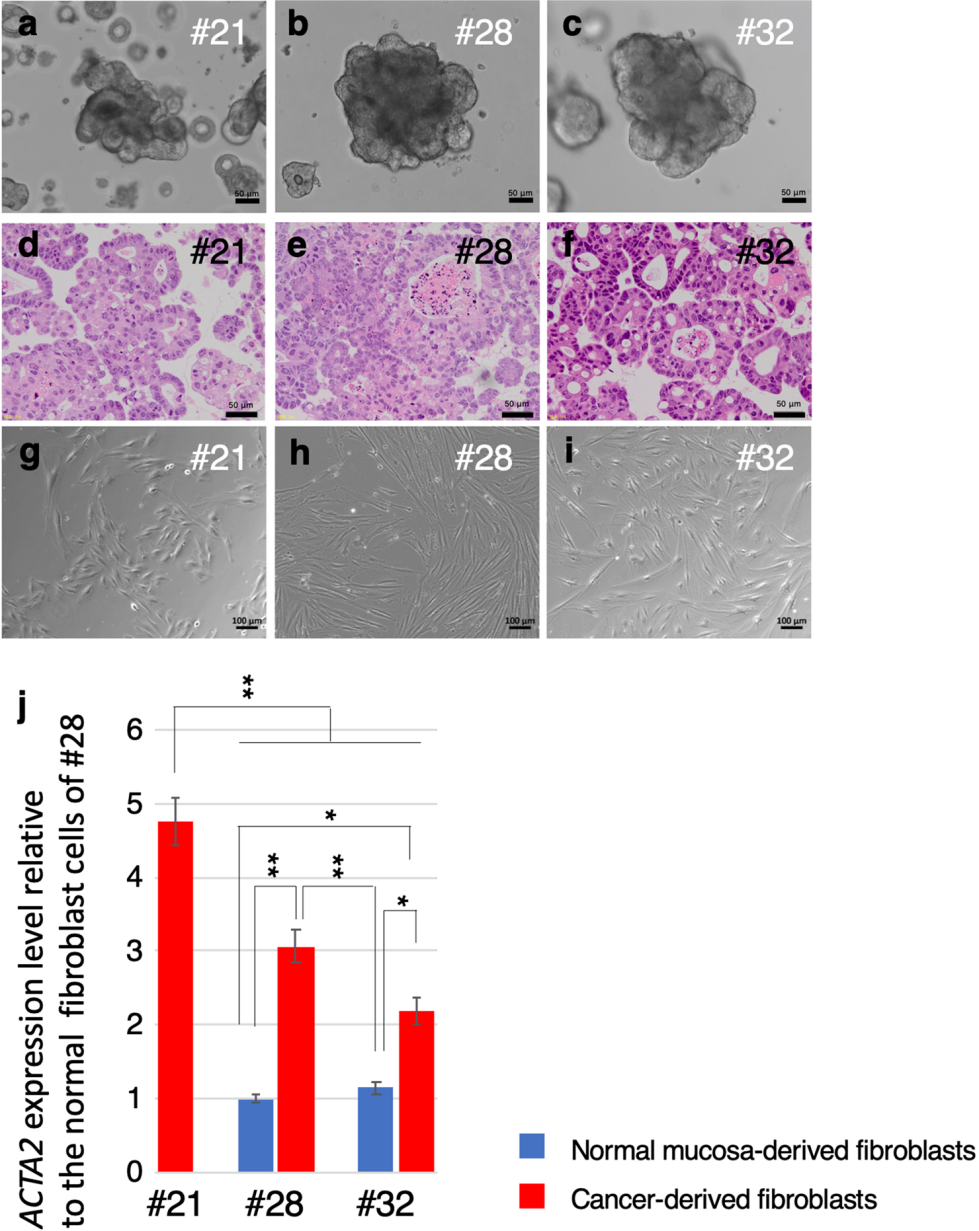
Morphological and gene expression features of CRC organoids and CAFs. (**a**–**c**) Bright-field microscopy images of CRC organoids. Scale bar, 50 μm. (**d**–**f**) Representative H&E micrographs of CRC organoids. Scale bar, 50 μm. (**g**–**i**) Bright-field microscopy images of fibroblasts established from CRC. Scale bar, 100 μm. (**j**) Increased expression levels of *ACTA2* in CAFs are shown by comparing with NFs. Blue and red bars represent the relative expression level of *ACTA2* in CAFs and NFs, respectively. The data represent the average of three independent experiments, each with three technical replicates. Error bars indicate ± s.d. *P < 0.05, **P < 0.01.

Organoids from 11/45 (#1, #11, #13, #16, #18, #21, #25, #28, #32, #33, and #44) cases were established. Wnt activators (Wnt3a/R-spondin 1) were not essential for all organoids and the p38 inhibitor (SB202190) enabled efficient establishment in 4/11 cases (Supplementary Table S1). CRC organoids had globular and/or irregularly elongated crypts with monolayered or partially multilayered structures (Fig. 1a–f). CRC-derived cancer associated fibroblasts (CAFs) (Fig. 1g–i) and normal mucosa-derived fibroblasts (NFs) were isolated from cancer tissues and adjacent normal tissues, respectively. The success rate of the presently established organoids was 24.4% (11/45), that of CAFs was 77.1% (27/35), and that of NFs was 88.6% (31/35). CAFs were established from cases obtained during the period from September 2016 to February 2018. The success rate of CRC-derived organoids did not correlate with the basic patient or pathological information, e.g., the sex, age of patient, or pathological stage of cancer (Supplementary Table S2). High expression of *ACTA2*, a marker of activated myofibroblasts and CAFs^13^ was observed in CAFs from cases #28 and #32, in comparison with the matched NFs (Fig. 1j). In case #21, NFs were not available; however, the expression level of *ACTA2* in CAFs was even higher than that in CAFs from #28 and #32.

### Mutation analysis by NCC Oncopanel

**Figure 2:**
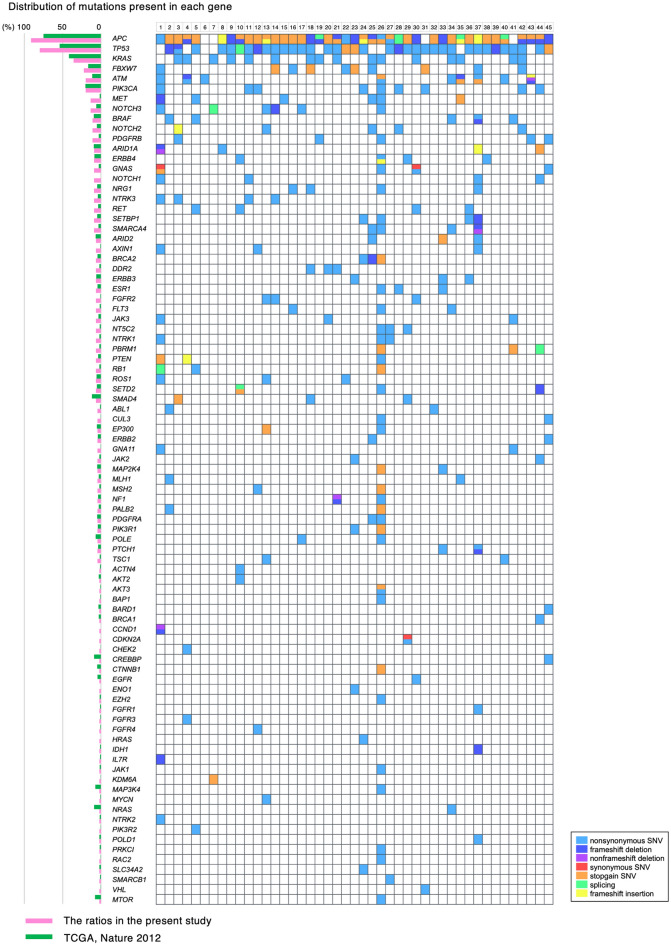
Comparison chart of gene mutations in 45 CRC samples. Mutations of 114 cancer-related genes were analyzed using NCC Oncopanel test. The left graph shows the mutation frequency of each gene in the present 45 samples (pink) and the TCGA data set (green). Most frequently mutated gene was *APC* followed by *TP53* and *KRAS*. Light blue; nonsynonymous SNV, blue; frameshift deletion, purple; nonframeshift deletion, red; synonymous SNV, orange; stopgain SNV, green; splicing, yellow; frameshift insertion.

Mutations in original CRC tissues and established organoids were analyzed by targeted sequencing using the NCC Oncopanel test. The prevalence rates of *APC, TP53, and KRAS* mutations in original tissues were 91.1%, 80.0%, and 35.6%, respectively (Fig. 2). The frequently mutated genes including *APC*, *TP53*, and *KRAS* were highly consistent between our cohort and TCGA (The Cancer Genome Atlas) database^24^. Most of the mutations found in the CRC tissues were maintained in their organoids with higher mutation allele frequencies. However, there are some exceptions, suggesting a reflection of intratumor heterogeneity. Small to large decreases, e.g., *MAP2K4* in #33, in the frequency of minor mutations in organoids were considered to be partly due to the clonal evolution of cancer cells during organoid culture (Table 1).

**Table 1:**
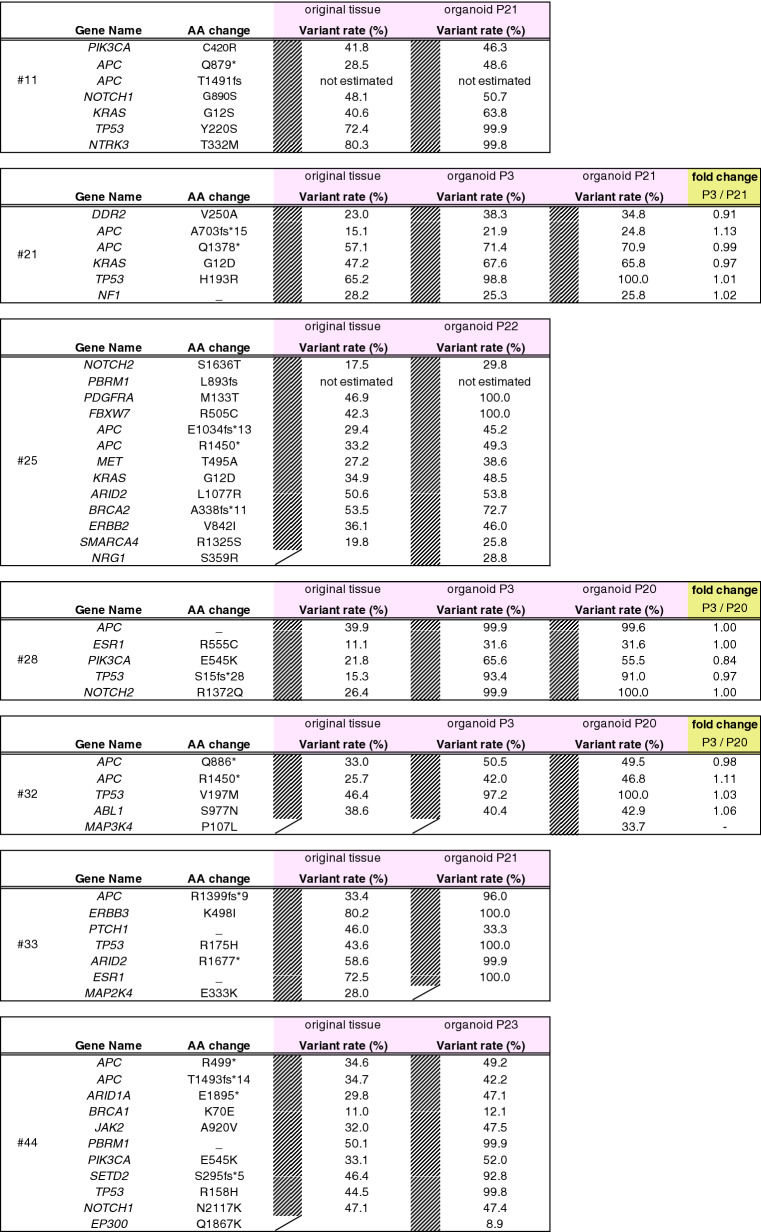
Comparison of variant rates of cancer-associated genes between original tumor tissues and organoids of principal 7 cases.

Then, we performed Sanger sequencing to confirm that the established fibroblasts did not harbor the mutations found in cancer tissues. The *TP53* mutations of the cases #21, #28, and #32 were detected in their organoids but not in their fibroblasts (Supplementary Fig. S2). Other mutations such as those of *DDR2* in #21, *ESR1* in #28, and *APC* in #32, were also not detected in the corresponding fibroblasts (data not shown).

### Gene expression analysis by DNA microarray

**Figure 3:**
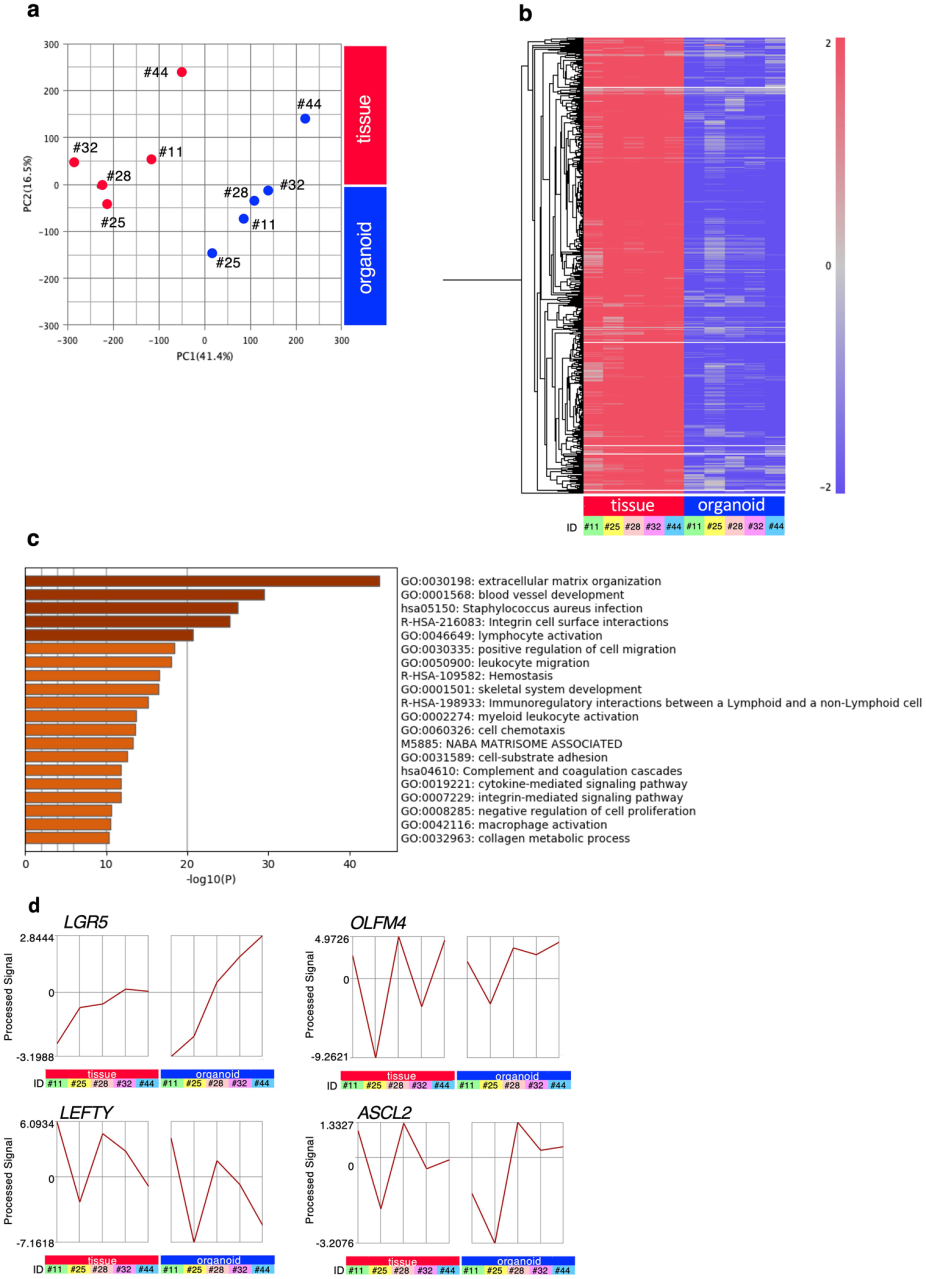
Comparative gene expression profile between original tumor tissues and organoids. PCA score plot displaying the distinct dissimilarities between the primary tumors and organoids from cases #11, #21, #25, #28, #32, and #44 (**a**). The PCA scores for components 1 and 2 were determined from the entire spectra. (**b**) In total, 586 probes with a gene expression profile similar to that with high expression only in tumor tissues in PC1 were extracted (Pearson’s correlation between 0.95 and 1). Tree clustering of these genes is shown. The clustering separated each sample type. (**c**) Gene Ontology analysis and significantly enriched GO terms of 586 probes whose expression in organoids was lower than that in the original tumors. (**d**) Expression profiles of intestinal stem cell marker genes. Line graphs using normalized microarray data show expression levels of *LGR5,* *OLFM4, LEFTY,* and *ASCL2.*

Gene expression analyses by DNA microarray were carried out using original CRC specimens and organoids. Data sets for original tumor tissues and organoids from 5 cases were successfully adjusted and validated for principal components analysis (PCA). The PC1-axis demonstrated a different spatial distribution between the original tumors and organoids. The two groups had significant differences in gene expression distribution, indicating two transcriptionally distant populations (Fig. 3a). There were two typical gene expression profiles of PC1 genes: One was the gene groups highly expressed in organoids and the other was gene groups whose expression in organoids was lower than that in the original tumors. In total, 586 probes exhibited a similar gene expression profile with the latter group of PC1 genes (Pearson’s correlation between 0.95 and 1). Based on Gene Ontology (GO) term analysis, the genes with lower expression in organoids than in the original tumors were enriched for GO terms “extracellular matrix organization”, “blood vessel development”, and “lymphocyte activation” (Fig. 3c). This reflected the lack of fibrous, vascular, and immune cell populations in organoids, whereas the original tumors consist of both epithelial cells and stromal cells. On the other hand, expression profiles of intestinal epithelial-stemness-related genes demonstrated that the organoids maintained the characteristics of the original CRC tissues (Fig. 3d). It is also possible that there was a subset of genes induced via cell–cell communication with other cell types, including CAFs.

### Cell proliferation abilities of CRC organoids were stimulated by co-culturing with paired CAFs

**Figure 4:**
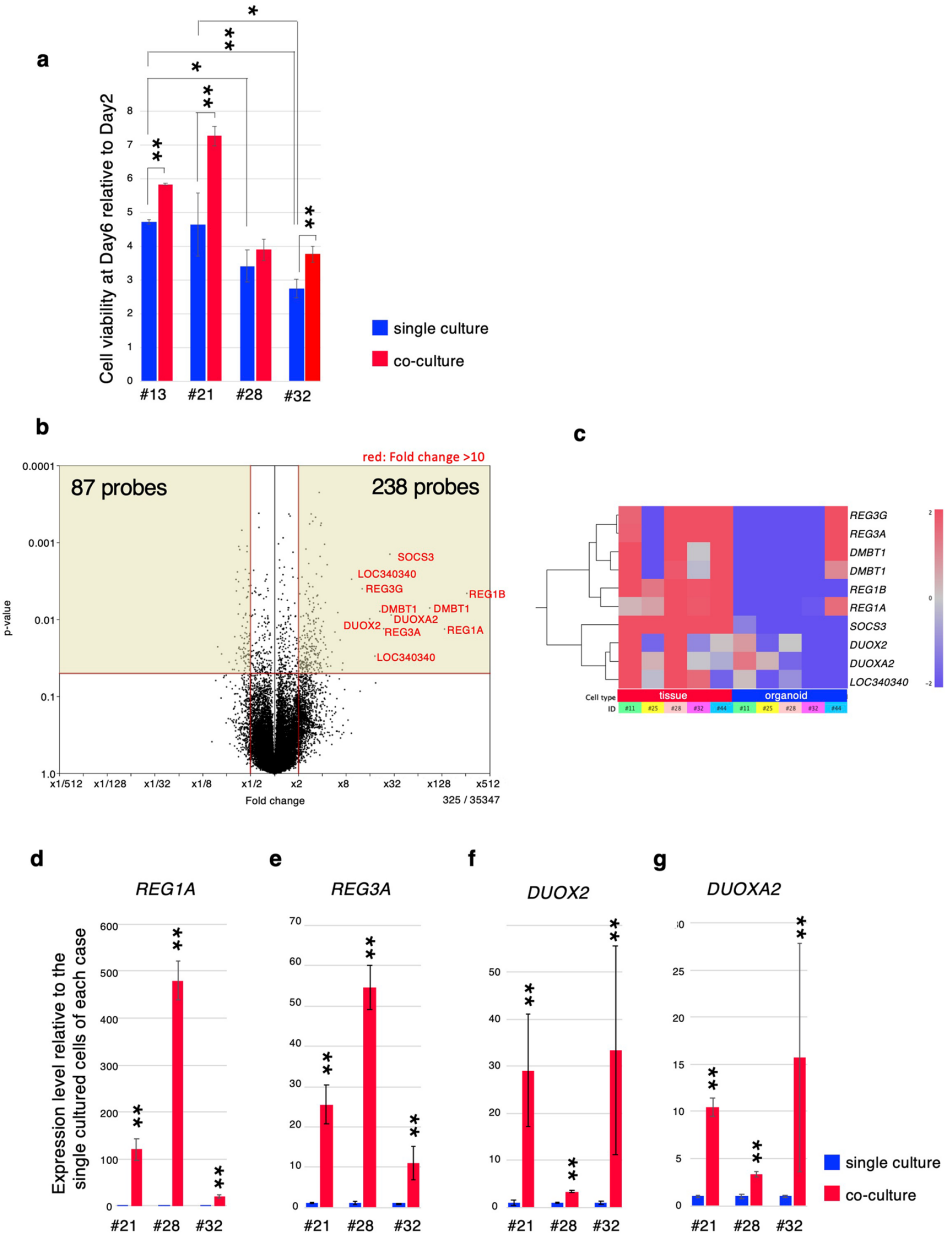
Comparison of cell viability and gene expression profile between organoids with or without co-culture with CAFs. Cell viability of single-culture and co-cultured organoids at Day 6. (**b**) Volcano plot of gene expression levels comparing those in single-culture organoids and those in organoids co-cultured with CAFs (n = 3 biological replicates). The y-axis shows the *P*-value for the differences in gene expression levels between organoids with and without co-culture with CAFs by a negative logarithm. The x-axis is the log2 difference in the estimated relative gene expression values. Vertical red lines represent the threshold of the log twofold change (equivalent to a twofold change). Thus, the dots in the beige-colored region correspond to genes that show a significant (*P* ≤ 0.05) twofold or greater change in gene expression between single-culture organoids and organoids co-cultured with CAFs. (**c**) Gene expression profile of CAF-induced genes with tenfold upregulation, including *REG* family genes and *DUOX* gene families, were extracted from microarray data for CRC tissues and single-culture organoids. Relative quantification of two CAF-induced gene group candidates*, REG1A* (**d**), *REG3A* (**e**), *DUOX2* (**f**), and *DUOXA2*, (**g**) measured by qRT-PCR. The expression levels of each genes were calculated by comparing with beta-*ACTIN*. Blue bars represent gene expression levels in organoids without CAFs. Red bars represent gene expression levels in organoids co-cultured with CAFs. The qRT-PCR data represent the average of three independent experiments, each with three technical replicates. Error bars indicate ± s.d. **P < 0.01.

The cell proliferation rate significantly varied among CRC organoid lines (Fig. 4a). CAFs were also established from the same CRC specimens in some cases. To address the effects of interaction between CRC organoids and corresponding CAFs, we developed a novel co-culture method using a chamber system. By co-culturing organoids with CAFs, cell viability of organoids increased by 1.2 to 1.5-fold compared with corresponding single-culture organoids in three out of four cases (Fig. 4a). This suggested that the tumor microenvironment constructed by effects of signaling between tumor cells and CAFs plays a role in the cell proliferative/anti-apoptotic abilities of tumor cells.

### Identification of upregulated genes by co-culturing with CAFs

To identify genes whose expression levels were different between organoids co-cultured with and without CAFs, we performed DNA microarray analysis. Volcano plot analysis demonstrated that the expression levels of 73 genes with 87 probes in organoids were reduced to less than half, whereas those of 177 genes with 238 probes were increased by more than double by co-culturing with CAFs (*P* < 0.05; Fig. 4b, Supplementary Tables S3, S4). These upregulated genes included *REG1A, REG1B, REG3A, REG3G, DMBT1, DUOXA2, DUOX2, SOCS3,* and *LOC340340,* with more than tenfold upregulation. Hierarchical clustering analysis confirmed that these CAF-induced genes were expressed at higher levels also in the original CRC tissues but not in single-culture organoids (Fig. 4c). To validate the microarray data obtained from the co-culture of CRC organoids and CAFs, two candidates for CAF-induced gene groups, *REG1A, REG3A, DUOX2*, and *DUOX2A,* were quantified by quantitative RT-PCR (Fig. 4d–g). The levels of *REG1A* increased by more than 121 (#21), 480 (#28), and 21-fold (#32) when co-cultured with CAFs. The levels of *REG3A* increased by more than 26 (#21), 55 (#28), and 11-fold (#32). The levels of *DUOX2* increased by more than 29 (#21), 3 (#28), and 33-fold (#32). The levels of *DUOXA2* increased by more than 10 (#21), 3 (#28), and 15-fold (#32). This analysis confirmed that the microarray data is reliable, and the degree of induction of each *REG* family and dual oxidase gene by CAFs varied among cases.

Other than *REG* family and dual oxidase genes, microarray analysis suggested that expression of *CEACAM6* and *CEACAM7*, members of the carcinoembryonic antigen-related cell adhesion molecule family, and *MUC1*, which inhibits the anti-tumor immune response, was upregulated by more than twofold (Supplementary Table S3).

Although many cancer-related genes were induced by the co-culture with CAFs, the degree of their induction by CAFs varied among cases.

GO term enrichment analysis for upregulated genes in organoids by co-culturing with CAFs revealed that innate immune response, including “cell wall disruption in other organisms”, “response to bacterium”, “complement and coagulation cascades”, and “interferon-gamma-mediated signaling pathway”, were significantly enriched (Supplementary Fig. S3a). These induced pathways by CAFs were abundantly expressed in CRC tissues but downregulated in single-culture CRC organoids (Supplementary Fig. S3b).

### CAFs derived from different cases exhibit case-specific ability for *REG1A* induction in organoids

**Figure 5:**
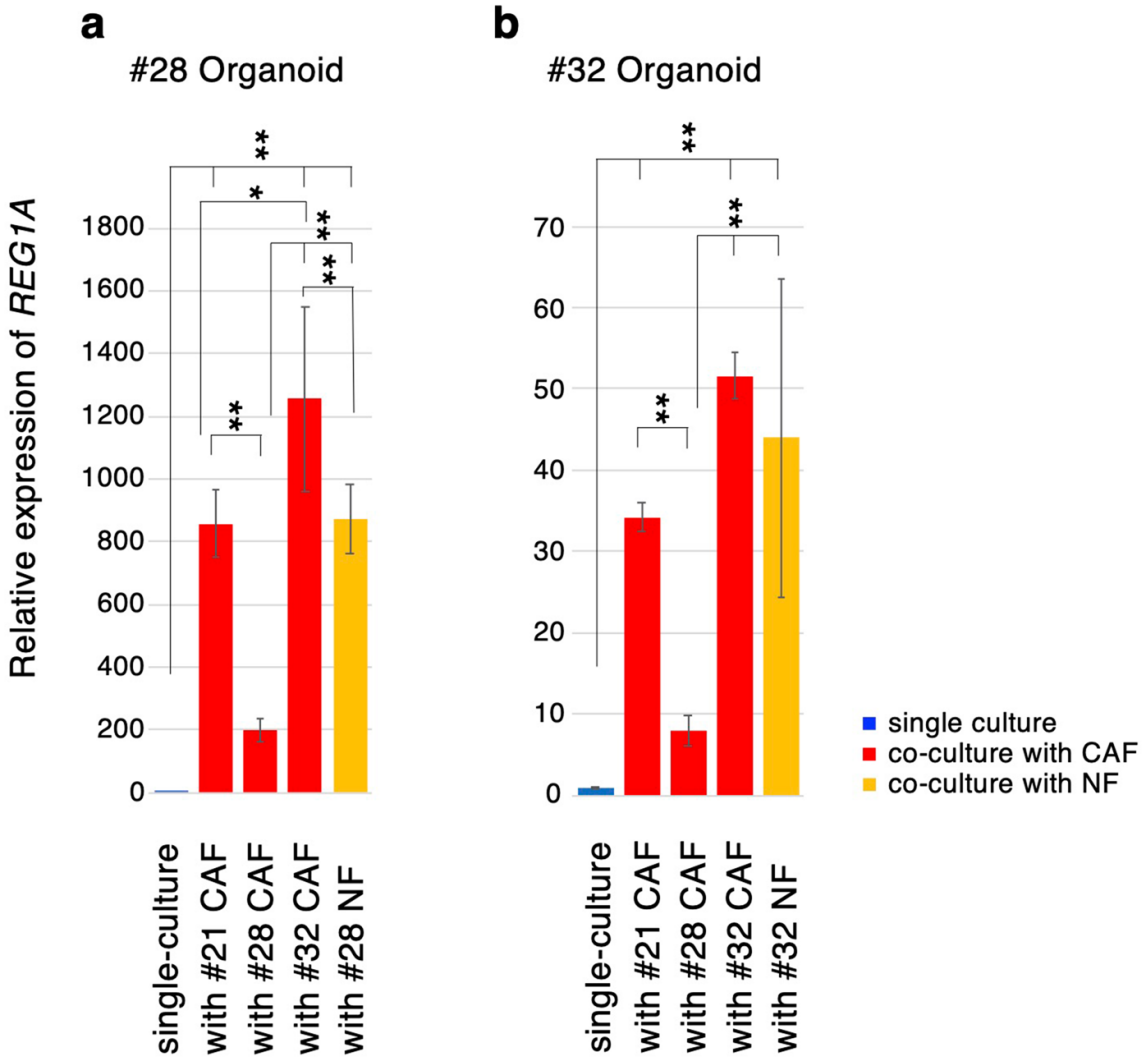
Induction of *REG1A* in organoids by co-culture with CAFs from three different cases and normal fibroblasts from one case. qRT-PCR for *REG1A* gene in organoids co-cultured with CAFs from three different cases and normal mucosa-derived fibroblasts was performed using #28 (**a**) and #32 organoids (**b**). Relative gene expression levels are shown with the expression level of single-culture organoids set to 1. The data represent the average of three independent experiments, each with three technical replicates. Error bars indicate ± s.d. *P < 0.05, **P < 0.01.

Organoids derived from different cases exhibited varying degrees of induction of *REG* family and dual oxidase genes by co-culturing with paired CAFs, e.g., organoids from cases #21 and 32 significantly induced dual oxidase genes, and those from case #28 significantly induced *REG* family genes (Fig. 4d–g). Next, combinations of organoids and CAFs derived from different cases were examined because CAFs consist of numerous cell types and their characteristics vary among lines^25,26^. Organoids of cases #28 and #32 co-cultured with CAFs from three different cases and corresponding normal mucosa-derived fibroblasts (NFs) exhibited almost identical induction patterns by co-culture with CAFs from each case and corresponding NFs (Fig. 5a,b), suggesting the case-specific ability of CAFs for the induction of *REG1A* in organoids. Thus, re-expressed genes in CRC organoids by co-culturing with CAFs may depend on the characteristics of each organoid and CAF line.

## Discussion

We established 11 organoids from 45 CRC cases, and simultaneously prepared paired CAFs and normal fibroblasts from several of them. The rate of generated CRC organoids in the present study was lower than those in previous reports^27,28^. Fujii et al. noted the following as causes of establishment failure of CRC organoids^27^: One possibility was difficulty in enzymatic digestion of carcinoma tissues and another was to be culture conditions, including not only the composition of culture medium, but also atmospheric oxygen concentrations. Therefore, further digestion methods and combination culture conditions should be considered to improve the rate of generation of CRC organoids.

Interactions between cancer cells and CAFs using colon or lung cancer cell line-originated organoids and patient-derived CAFs were previously reported^22,23^. Other reports suggested that the molecular characteristics of CAFs vary among cases^29,30^. The newly established CRC model of organoids co-cultured with paired CAFs from the same case in the present study may provide novel insights into effects of signaling between these cell types.

Target sequencing analyses of original tumor tissues revealed that mutation patterns varied among patients, and the most frequently mutated genes were *APC, TP53,* and *KRAS,* as previously reported^24^. In addition, most mutations found in the original tumor tissues were conserved in organoids^31^. The mutation rates in CRC organoids were higher than those in CRC tissues, with several exceptions (Table 1). This may be because CRC organoids consist of only epithelial tumor cells, whereas CRC tissues include epithelial tumor cells and stromal cells, which do not have mutations. Indeed, CAFs did not carry the mutations found in corresponding CRC organoids (Supplementary Fig. S2). The fold changes in the mutation ratios (early passage/late passage—up to passage 20) were almost identical (0.84–1.13) (Table 1), as previously reported in ovarian cancer organoids^32^. This suggests that organoids from CRC passaged up to 20 times can be applied as an ex vivo model in basic and translational studies for the evaluation of anti-cancer drugs. Furthermore, cancer stem cells comprising CRC organoids and their proliferative ability were maintained in our culture conditions.

Next, to examine whether CRC organoids can reproduce the gene expression of the original tumor tissues, gene expression profiles were compared between the original tumor tissues and established CRC organoids using DNA microarray (Fig. 3). As a result, CRC organoids lacked gene expression from mesenchymal cell populations and blood cells, whereas each CRC organoid had a variable expression profile for intestinal stem cell marker genes, e.g. *LGR5* and *OLFM4*, as observed in CRC tissues. This suggests that gene expression in CRC organoids is mostly conserved, but they lack cell–cell communication with the tumor microenvironment, including fibroblasts and immune cells.

In this study, a co-culture system using the CRC organoids and paired CAFs and/or normal fibroblasts from each case was prepared, and gene expression profiles for both CRC organoids with or without co-culture with CAFs were compared. As a result, we identified 177 genes, including *REG* family and *DUOX* family genes, which were markedly upregulated by more than twofold by co-culturing with CAFs in all three cases analyzed. Some of these upregulated genes were reported to have oncogenic functions. For example, *REG* family genes are known to be upregulated in several carcinomas compared with normal tissue^33–36^. *REG* family genes have cell-proliferative and anti-apoptotic functions^37^. *REG3A* is also known to act as an extracellular matrix (ECM)-targeted scavenger of reactive oxygen species (ROS) in a dose-dependent manner and to prevent ROS-induced mitochondrial damage due to acetaminophen overdose^38,39^. ROS is a key regulator for EMT, and is produced by *NOX* gene family and *DUOX* family genes^40^. Therefore, *REG3A* and *DUOX2* gene expression is considered to be important for EMT. *DUOX* family genes also have cell-proliferative functions^40,41^. Moreover, *DUOX2* was reported to have anti-cancer drug resistant activities through EMT^42^. GO term enrichment analysis for the above mentioned 177 upregulated genes revealed that co-culturing of CRC organoids with CAFs induced innate immune responses, suggesting that some oncogenic signal cascades induced by cell–cell interaction between organoids and CAFs were mediated by the signal pathways related to immune responses.

Other than immune response- and external stimulus-related genes, *CEACAM6*, a member of the carcinoembryonic antigen (CEA) family^43^, was also upregulated by more than twofold in CRC organoids by co-culturing with CAFs. CEA is normally produced in gastrointestinal tissue during fetal development, but its production stops before birth. Consequently, CEA is usually present at low levels in the blood of healthy adults (approximately 2–4 ng/ml)^44^. However, the serum levels increase in some types of cancer, suggesting its utility as a diagnostic marker and/or a drug efficacy marker in clinical tests. *CEACAM6* was highly upregulated in colon cancer tissues and may therefore be a suitable candidate for a diagnostic marker of colorectal cancer^45^. *CEACAM6* loss increases mitochondrial basal and maximal respiratory capacity. It also affects several hallmarks of pancreatic ductal adenocarcinoma (PDA), including fibrotic reactions, immune regulation, and energy metabolism, and was recently investigated as a novel therapeutic target in PDA^46^. Thus, there is a subset of genes expressed in CRC tumor cells in the original tissues, but not in the CRC organoids without co-culture with CAFs. These silenced genes in CRC organoids were induced by the co-culture with CAFs (Fig. 4c).

The degrees of upregulation of the genes by co-culturing with CAFs varied among the CRC cases. We investigated whether these effects on the gene expression changes depend on the ability of CAFs or their compatibility with CRC organoids. Organoids from two CRC cases exhibited almost identical induction patterns by co-culture with CAFs from three different cases and paired normal mucosa-derived fibroblasts (Fig. 5). Therefore, the degrees of upregulation of several genes, e.g., *REG1A,* depend on the ability of CAFs from CRC. In addition, normal fibroblasts induced *REG1A* expression, suggesting that some factors, possibly growth factors/cytokines secreted by CRC organoids or miRNA delivered by extracellular vesicles (EVs) secreted from CRC organoids, can induce the expression of certain genes to a similar degree as CAFs. To address which factors are essential for the induction of genes in CRC organoids, detailed analyses of the co-culture system of CRC organoids are needed.

The upregulated genes mentioned above were related to malignancy of tumor cells, and the malignancy of cancer may partly depend on the characteristics of CAFs and/or original normal fibroblasts. Indeed, cell viability of organoids increased by 1.2 to 1.5-fold compared with corresponding single-culture organoids in three of four cases by co-culturing with CAFs (Fig. 4a); however, those in one case (#28) were not altered. We compared the efficiency of induction of the *REG1A* gene in organoids by co-culture with CAFs from three cases and it was the lowest by #28 CAFs.

In conclusion, (1) gene mutation patterns, fold changes in the mutation allele frequencies, and expression profiles for intestinal stem cell marker genes were nearly similarly maintained in CRC organoids as in the original tumor tissues, suggesting that CRC organoids can be applied as an ex vivo model in basic and translational studies. (2) Expression levels of several genes, which are highly expressed in original CRC tissues, were downregulated in organoids but re-expressed by co-culturing with CAFs. They comprised immune response- and external stimulus-related genes, e.g., *REG* family and dual oxidases (*DUOX*s). In addition, gene induction by co-culturing with CAFs varied depending on the CAFs from each CRC case, suggesting that the present co-culture system of CRC organoids with paired CAFs partially reproduces the tumor-microenvironment.

## Methods

### Tissue sampling

A total of 45 surgical specimens of colorectal carcinomas (CRCs) and adjacent normal mucosa were obtained between February 2016 and February 2018 at National Cancer Center Hospital. The clinical data and samples were handled in accordance with relevant domestic guidelines and regulations in Japan. The use of patients’ surgical specimens in this study was approved by the ethics committee of the National Cancer Center, Tokyo, Japan (2015-108), and written informed consent was obtained from all patients. After sampling for pathological evaluation, they were stored on ice, and each sample was dissected into approximately 2–5-mm cubes and used for culture of organoids and fibroblasts. Remaining fragments were simultaneously frozen in liquid nitrogen and stored at − 80 °C for isolation of DNA and RNA, or fixed with 10% buffered formalin for preparation of tissue sections for morphological identification of the organoid- and fibroblast-originated tumor tissues. The fragments for RNA isolation were stored in RNA*later* solution (ThermoFisher Scientific, Tokyo, Japan) at 4 °C overnight before storage.

### Organoid culture

The protocol employed for organoid culture was a modified version of those previously reported^27,47^. The fragments of CRC tissues were washed with cold HBSS(−), minced with scissors, and washed again. These fragments were incubated in Accumax (Innovative Cell Technologies, San Diego, CA, USA) at room temperature or TrypLE Express (ThermoFisher Scientific, Tokyo, Japan) at 37 °C for 30 min with shaking. The digested fragments were rinsed with cold HBSS(−), separated using a 100-μm cell strainer, and pelleted. Advanced DMEM/F12 (ThermoFisher Scientific) supplemented with 1× penicillin–streptomycin, 500 ng/ml of Amphotericin B (FUJIFILM Wako, Osaka, Japan), 1× Zell Shield (Minelva Biolabs GmbH, Berlin, Germany), 10 mM HEPES (ThermoFisher Scientific), 1× l-glutamine (FUJIFILM Wako), [Leu^15^]-GastrinI (Sigma-Aldrich, Tokyo, Japan), 1 mM N-acetyl-l-cysteine (FUJIFILM Wako), 1× B27 supplement (ThermoFisher Scientific), 1% BSA (FUJIFILM Wako), and 10 μM Y27632 (FUJIFILM Wako) was used as the basal culture medium for CRC organoids. The pellet was suspended in the basal culture medium containing the following factors: 50 ng/ml of Recombinant Human EGF (Peprotech, Rocky Hill, NJ, USA), 100 ng/ml of Noggin (Peprotech), and 500 nM A83-01 (FUJIFILM Wako).

In a 12-well plate, 65 μl of Matrigel (Corning, Bedford, MA, USA)/well was polymerized for 15 min at 37 °C and a 650-μl cell suspension/well × 3 was seeded and incubated in a 37 °C humidified CO_2_ incubator. After 24 h, the supernatants were removed and 85 μl/well of Matrigel was overlaid. After Matrigel polymerization, the basal culture media containing different factor combinations [A:250 ng/ml of R-spondin 1 (Peprotech) + 20 ng/ml of Wnt-3a (Peprotech) + 25 μM SB202190, B: 25 μM SB202190, and C: none] was used to select the most efficient growth media during several passages. The organoids were dispersed by Accumax and passaged approximately once a week. Zell Shield was not used after several passages. When organoids with more than 10 passages (P10) survived after a freeze and thaw cycle, they were defined as “successfully established”.

### Fibroblast culture

Two to three tissue fragments were washed three times with HBSS(–), placed in a 60-mm dish, and minced with scissors. Then, culture medium, RPMI-1640, containing l-Glutamine (FUJIFILM Wako) and 10% FBS, penicillin–streptomycin was added into the dish and cells were incubated at 37 °C in a humidified 5% CO_2_ incubator. After they reached 70% confluency, cells were passaged using TrypLE Express dissociation reagent (ThermoFisher Scientific).

### Co-culture of organoids with fibroblasts using a chamber system

Organoids were cultured in the cell culture inserts with a porous membrane and fibroblasts were cultured in the carrier plates (Corning). The pore size of the insert was 1.0 μm to allow the free exchange of media but not cells to migrate through. One day before starting the co-culture, fibroblasts were dissociated into single cells using TrypLE Express and 1 × 10^4^ cells were cultured in a 24-well plate with RPMI-1640 containing penicillin–streptomycin, Amphotericin B, and 10% FBS. For organoid culture, cell culture inserts were set on the 24-well companion plate and 20 μl of Matrigel was polymerized on the insert. Organoids were dissociated by Accumax and resuspended in optimized media for organoids described above. The cell suspension (1 × 10^4^ cells/200 μl) was seeded onto the Matrigel and 620 μl of media for organoids was added to the basal compartment. Fibroblasts and organoids were incubated at 37 °C in a CO_2_ incubator.

The next day, apical and basal media were removed from the plate containing organoids attached on the Matrigel, and organoids were covered with 20 μl of Matrigel and polymerized at 37 °C for 15 min. The inserts containing organoids were transferred to the fibroblast-containing compartment plate after changing the medium from that for fibroblasts to 820 μl of optimized media for organoids. The co-culture plate was incubated at 37 °C in a CO_2_ incubator for 96 h, and organoids and fibroblasts were collected separately for gene expression analysis.

### Targeted sequencing analysis

Genomic DNA from 45 samples of CRC and organoids of cases #11, #21, #25, #28, #32, #33, and #44 were prepared using NucleoSpin Tissue kit (Takara Bio, Kusatsu, Japan) according to the manufacturer’s protocol. Targeted sequencing analyses of those DNAs were performed using the NCC Oncopanel v4 test, which can analyze mutations of 114 genes and amplifications and fusions of 12 genes^48^. Procedures for targeted sequencing and data analysis were previously described^48^.

*TP53* mutations identified by the NCC Oncopanel test were confirmed by Sanger sequencing. The PCR products including mutations were amplified using specific PCR primers. Primers used for A578G and G589A mutations of #21 and #32, respectively, were forward: 5′-GGAGGTCAAATAAGCAGCAGG-3′ and reverse: 5′-GGCCTCTGATTCCTCACTGA-3′. Primers used for a 45_48TCAG deletion of #28 were forward: 5′-CCCAACCCTTGTCCTTACCA-3′ and reverse: 5′-CAGTCAGATCCTAGCGTCGA-3′. The amplified PCR products were directly sequenced by Sanger sequencing using the following primers. The primer for #21 and #32 was 5′-ACAACCACCCTTAACCCCTC-3′. The primer for #28 was 5′-CCCAACCCTTGTCCTTACCA-3’.

### Gene expression analysis using DNA microarray

Total RNA was extracted from original tumor tissues and established organoids of cases #11, #25, #28, #32, and #44 using NucleoSpin RNA Plus (TaKaRa) according to the manufacturer’s protocol. Total RNA was extracted from three replicates of co-cultured organoids and fibroblasts for #21, #28, and #32 using the RNeasy Micro kit (QIAGEN, Tokyo, Japan). The quality of the RNA samples was evaluated using an Agilent 2100 Bioanalyzer (Agilent Technologies, Santa Clara, CA, USA) and highly qualified RNA samples with a RIN score > 7.0 were selected for further DNA microarray analysis. Triplicate samples were used as an RNA cocktail. Cy3-labeled cRNA was hybridized on SurePrint G3 Human GE Microarray GE 8 × 60 K Ver.3.0 (Agilent Technologies) according to the manufacturer’s instructions. The Subio Platform, Subio Basic plug-in, and Subio Advanced plug-in (ver 1.24) (Subio, Kagoshima, Japan) were used for data analysis.

### Quantitative real-time PCR

One µg of total RNA for each sample was treated with DNase I (NIPPON GENE, Tokyo, Japan) prior to reverse transcription to cDNA using Multiscribe Reverse Transcriptase with random primers (ThermoFisher Scientific). qRT-PCR was performed with SsoAdvanced Universal SYBR Green Supermix (BIO-RAD, California, USA) using DNA Engine Opticon 2 (MJ Research, Quebec, Canada). Reactions were run in triplicate in three independent experiments. Data were normalized with the housekeeping gene β-*ACTIN* and were calculated by the 2-ΔΔCT method^49^. Data were presented as means ± SD (1.0-fold as the control). The primer sequences were as follows: β*-ACTIN* forward 5′-AAACTGGAACGGTGAAGGTG-3′ and reverse AGAGAAGTGGGGTGGCTTTT3′, *ACTA2* forward 5′-CTGTTCCAGCCATCCTTCAT-3′ and reverse 5′-GCTGGAAGGTGGACAGAGAG-3′, *REG1A* forward 5′-CTGGAATCCTGTGCTTGAGG-3′ and reverse 5′-GGTCTCCTACAAGTCCTGGG-3′, *REG3A* forward 5′-CCTCTGGAAACCTGGTGTCT-3′ and reverse 5′-CCACTCCCAACCTTCTCCAT-3′, *DUOX2* forward 5′-GAGCCCTTCTTCAACTCCCT-3′ and reverse 5′-GGAGGACAGGCTCAGAAGTT-3′, and *DUOXA2* forward 5′-GGTCTCCTACAAGTCCTGGG-3′ and reverse 5′-TTTACAGATCGCCCCAGGAG-3′.

### Cell viability

The viability of organoids was assessed after co-culture with fibroblasts for 96 h using the chamber system. After aspirating culture medium, 100 μl of fresh culture medium was added. Then, Matrigel including organoids was scraped using a mini cell scraper, 100 μl of CellTiter-Glo 3D reagent was added (Promega, Tokyo, Japan), and organoids were disrupted by pipetting. Suspensions were transferred to 96-well assay plates and incubated at room temperature for 25 min. After incubation, the luminescence was measured using Synergy H1 (BioTek, Tokyo, Japan).

### Gene Ontology (GO) enrichment analysis

To clarify the biological meaning of the key modules, the gene information was loaded into Metascape (http://metascape.org) for Gene Ontology (GO) enrichment analysis^50^. Terms with a *P*-value < 0.01, a minimum count of 3, and an enrichment factor > 1.5 were collected and grouped into clusters based on their membership similarities (Kappa scores > 0.3).

### Statistical analysis

The associations between clinical factors and the establishment of organoids were tested using Fisher’s exact test in EZR^51^. For qRT-PCR and cell viability assay, results were presented as the mean ± s.d. Differences between groups were analyzed by the Student’s-t-test or one-way ANOVA using EZR. *P-*values of < 0.05 were considered significant.

## Supplementary Information

Supplementary Information.
