# Author Correction: Re-expression of *REG* family and *DUOX*s genes in CRC organoids by co-culturing with CAFs

**DOI:** 10.1038/s41598-021-93680-0

**Published:** 2021-07-29

**Authors:** Mie Naruse, Masako Ochiai, Shigeki Sekine, Hirokazu Taniguchi, Teruhiko Yoshida, Hitoshi Ichikawa, Hiromi Sakamoto, Takashi Kubo, Kenji Matsumoto, Atsushi Ochiai, Toshio Imai

**Affiliations:** 1 Central Animal Division, Fundamental Innovative Oncology Core, National Cancer Center Research Institute, 5-1-1 Tsukiji, Chuo-ku, Tokyo, 104-0045 Japan; 2 Department of Diagnostic Pathology, National Cancer Center Hospital, 5-1-1 Tsukiji, Chuo-ku, Tokyo, 104-0045 Japan; 3 Department of Clinical Genomics, Fundamental Innovative Oncology Core, National Cancer Center Research Institute, 5-1-1 Tsukiji, Chuo-ku, Tokyo, 104-0045 Japan; 4 Department of Allergy and Clinical Immunology, National Research Institute for Child Health and Development, 2-10-1 Okura, Setagaya-ku, Tokyo, 157-8535 Japan; 5 Exploratory Oncology Research and Clinical Trial Center, National Cancer Center, 6-5-1 Kashiwanoha, Kashiwa, Chiba 277-8577 Japan

Correction to: *Scientific Reports* 10.1038/s41598-021-81475-2, published online 22 January 2021

The original version of this Article contained errors in Table 1, where the AA change was omitted for a number of genes due to a technical error during gene mutation analysis. The correct and incorrect values appear below.

Incorrect: 
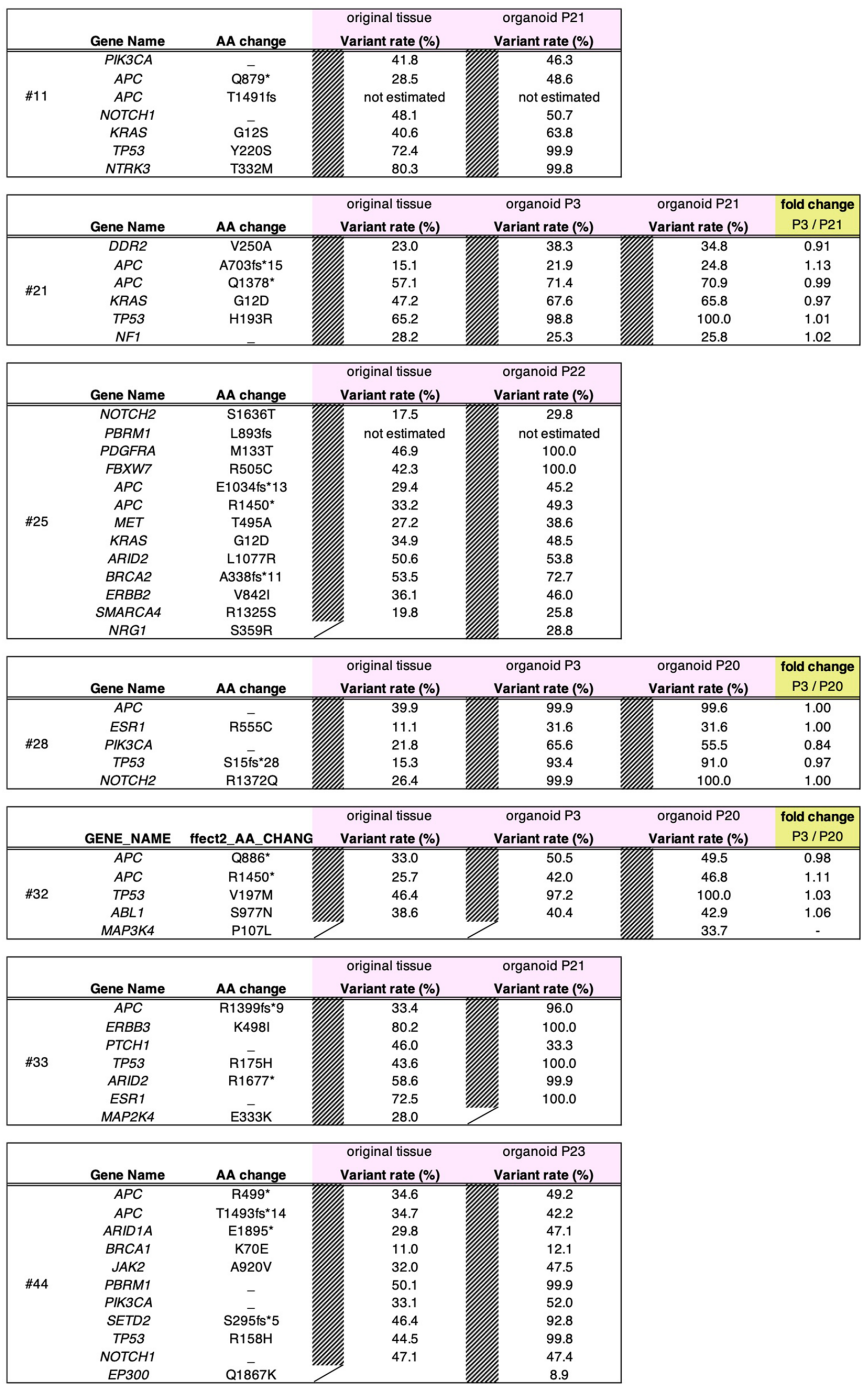


Correct:

**Table 1:**
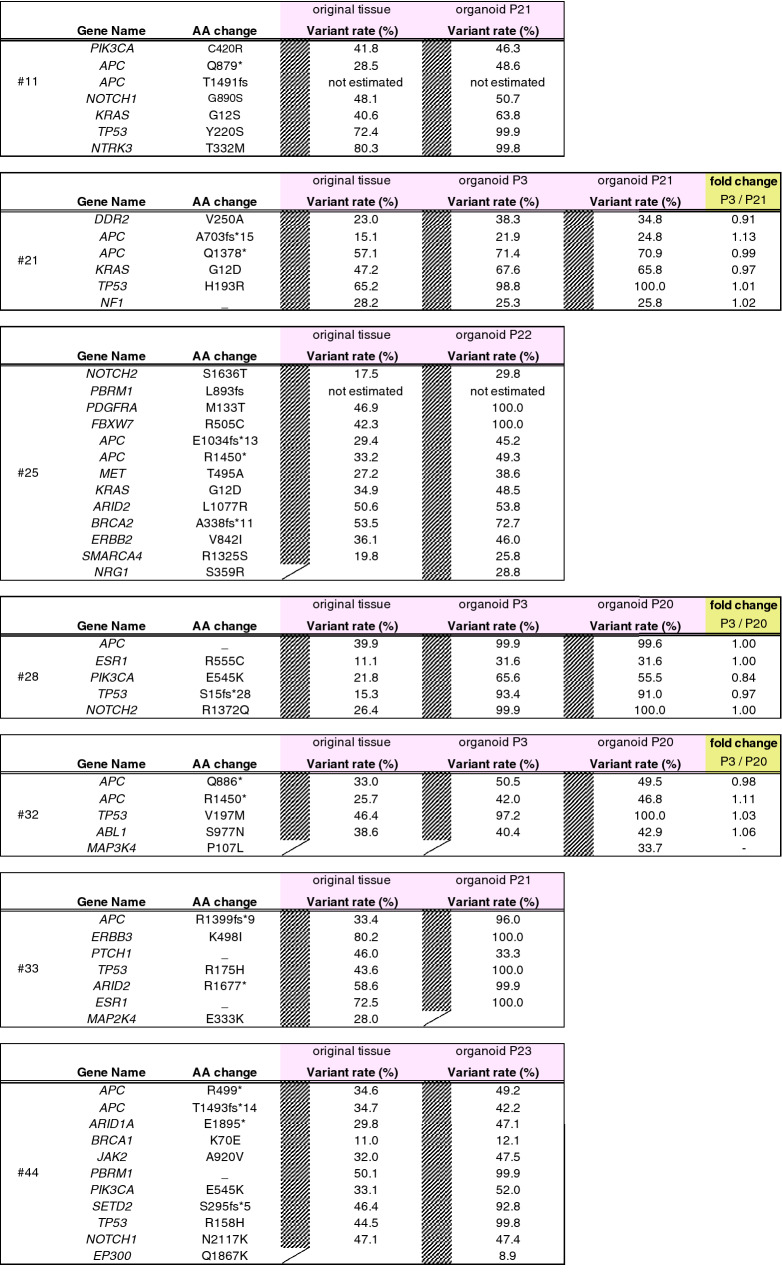
Comparison of variant rates of cancer-associated genes between original tumor tissues and organoids of principal 7 cases.

The original article has been corrected.

