# Supplementary material for: Re-expression of REG family and DUOXs genes in CRC organoids by co-culturing with CAFs
Source: Sci Rep. 2021 Jan 22;11:2077. doi: 10.1038/s41598-021-81475-2 (PMC7822883; doi:10.1038/s41598-021-81475-2)
Supplement: Supplementary file 1 — Supplementary Information. [file 41598_2021_81475_MOESM1_ESM.pdf]

Re-expression of *REG* family and *DUOX*s genes in CRC organoids by co-culturing with CAFs

Mie Naruse, Masako Ochiai, Shigeki Sekine, Hirokazu Taniguchi, Teruhiko Yoshida, Hitoshi Ichikawa, Hiromi Sakamoto, Takashi Kubo, Kenji Matsumoto, Atsushi Ochiai, Toshio Imai

Supplementary Table S1

Basic patient and pathological characteristics of 45 CRC patients.

Location of carcinoma: C’ Cecum; A’ Ascending colon; T’ Transverse colon; D’ Descending colon; S’ sigmoid colon; RS’ Rectosigmoid colon; Ra’ upper rectum; Rb’ lower rectum. Pathology of carcinoma: tub1’ well differentiated type; tub2’ Moderately differentiated type. Medium used for establishment: (A)’ the basal culture media + R-spondin 1 + Wnt-3a + SB202190; (B)’ the basal culture media + SB202190; (C)’ the basal culture media + none.

	Case#	Age	Sex	Location	Stage	TNM	Pathology	Metastasis	Organoid-T (Medium used for establishment)	Fibroblast-T
From February to March 2016	1	61	Male	S	Ⅱ	T3N0M0	tub2 +tub1	absent	established (C)	not done
	2	52	Male	S	Ⅳ	T4N1M1	tub2	present	failed	not done
	3	62	Female	S	Ⅱ	T3N0M0	tub2	absent	failed	not done
	4	66	Female	C	Ⅳ	T4N1M1	tub1 +tub2	present	failed	not done
	5	61	Male	Rs	Ⅲa	T3N1M0	tub1 +tub2	present	failed	not done
	6	51	Male	Rb	Ⅱ	T3N0M0	tub1 +tub2	absent	failed	not done
	7	57	Male	A	Ⅳ	T4N2M１	adenosquamous	present	failed	not done
	8	68	Male	Rb	Ⅱ	T3N0M0	tub2	absent	failed	not done
	9	44	Female	Rs	Ⅳ	T3N0M0	tub2	absent	failed	not done
From September to December 2016	10	65	Female	Ra	Ⅱ	T2N0M0	tub2 +tub1	absent	failed	established
	11	66	Male	D	Ⅱ	T4N0M0	tub1 +tub2	absent	established (C)	failed
	12	74	Female	Ra	Ⅱ	T3N0M0	tub1 +tub2	absent	failed	established
	13	40	Female	Rs	Ⅲb	T4N2M0	tub1 +tub2	present	established (B)	established
	14	50	Female	Rb	Ⅱ	T3N0M0	tub2 +tub1	absent	failed	established
	15	50	Female	D	Ⅱ	T3N0M0	tub1 +tub2	absent	failed	established
	16	64	Male	Rs	Ⅱ	T3N0M0	tub2 +tub1	absent	established (B)	established
	17	58	Female	S	Ⅱ	T3N0M0	tub2	absent	failed	established
	18	57	Male	Rs	Ⅱb	T4N2M0	tub2 +tub1	present	established (B)	failed
	19	64	Male	Rb	Ⅲa	T1N1M0	tub1 +tub2	present	failed	failed
	20	59	Female	Rs	Ⅲa	T3N1M0	tub1	present	failed	established
	21	61	Male	T	Ⅲa	T4N1M0	tub1 +tub2	present	established (C)	established
	22	53	Male	Rs	Ⅱ	T3N0M0	tub1 +tub2	absent	failed	failed
	23	70	Female	Rb	Ⅲa	T3N1M0	tub2	present	failed	established
	24	79	Male	Ra	Ⅲb	T4N2M0	tub1 +tub2	present	failed	established
	25	61	Male	Ra	Ⅲb	T4N2M0	tub2+por	present	established (B)	established
From August 2017 to February 2018	26	41	Male	Rb	IIIb	T3N3M0	muc	absent	failed	established
	27	68	Male	Rs	IIIa	T4bN1M0	tub1	absent	failed	established
	28	57	Male	S	IV	T3N0M1	tub1	present	established (C)	established
	29	79	Male	S	IIIa	T3N1M0	tub2	absent	failed	established
	30	37	Male	Rs	II	T3N0M0	tub1	absent	failed	established
	31	49	Female	S	I	T2N0M0	tub1	absent	failed	established
	32	61	Male	C	II	T3N0M0	tub1	absent	established (C)	established
	33	79	Male	Rb	I	T2N0M0	tub1	absent	established (C)	failed
	34	73	Female	Rs	II	T3N0M0	tub1	absent	failed	established
	35	74	Male	Rb	II	T3N0M0	muc	absent	failed	established
	36	87	Female	Rs	I	T2N0M0	tub1	absent	failed	established
	37	74	Female	C	IIIa	T3N1M0	tub1	absent	failed	established
	38	72	Male	S	IV	T3N2M1	tub1	present	failed	failed
	39	66	Male	S	IIIa	T3N1M0	tub1	absent	failed	failed
	40	66	Male	S	II	T3N0M0	tub1	absent	failed	established
	41	36	Female	Rs	IV	T3N0M1	tub2	present	failed	established
	42	60	Male	S	II	T2N0M0	tub1	absent	failed	established
	43	88	Male	A	II	T3N0M0	tub2	absent	failed	failed
	44	66	Female	A	II	T3N0M0	tub1	absent	established (C)	established
	45	41	Female	S	III	T3N0M0	tub2	absent	failed	established

Supplementary Table S2

The associations between the clinical factors and the establishment rates of organoids

Attribute	Patient/tumor features	Total number	Not established (n=33)	Established (n=11)	*p*-value
**Age**	>65	19	16 (84.2)	3 (15.8)	0.3093
	<65	26	18 (69.2)	8 (30.8)	
**Sex**	F	18	16 (88.9%)	2 (11.1 %)	0.1563
	M	27	18 (66.7%)	9 (33.3%)	
**Location**	L	38	30 (78.9%)	8 (21.1%)	0.3368
	R	7	4 (57.1%)	3 (42.9%)	
**Stage**	I or II	24	17 (70.8%)	7 (29.2%)	0.503
	III or IV	21	17 (81.0%)	4 (19.0%)	
**TNM**	T1 or T2	6	5 (83.3%)	1 (16.7%)	0.1205
	T3	29	24 (82.8%)	5 (17.2%)	
	T4	10	5 (50.0%)	5 (50.0%)	
**Pathology**	tub1	26	19 (73.1%)	7 (26.9%)	0.8762
	tub2	16	12 (75.0%)	4 (25%)	
	others	3	3 (100 %)	0 (0%)	
**Metastasis**	-	30	24 (80.0%)	6(20%)	0.4639
	+	15	10 (66.7%)	5 (33.3%)	
**RAS-PIK3CA**	mutant	35	17 (70.8%)	7 (29.2%)	0.503
	normal	10	17 (81.0%)	4 (19.0%)	
**Tp53**	mutant	35	26 (74.3%)	9 (25.7%)	1
	normal	10	8 (80.0%)	2 (20.0%)	

Supplementary Table S3

Upregulated genes in CRC organoids with co-culture with CAFs.

Microarray probes with *P*<0.05, Fold change>2, are shown.

Probe NAME	GB_ACC	GENE_SYMBOL	ENSEMBL_ID	P-Value	Fold Change
A_23_P389500	NM_006507	*REG1B*	ENST00000305089	0.005	264.151
A_23_P90743	NM_002909	*REG1A*	ENST00000233735	0.013	138.088
A_23_P86599	NM_007329	*DMBT1*	ENST00000338354	0.007	90.072
A_33_P3250443	NM_207581	*DUOXA2*	ENST00000491993	0.009	29.429
A_23_P207058	NM_003955	*SOCS3*	ENST00000330871	0.001	28.360
A_23_P119936	NM_138938	*REG3A*	ENST00000305165	0.013	23.685
A_33_P3300312	NM_007329	*DMBT1*	ENST00000359586	0.008	21.171
A_23_P151851	NM_014080	*DUOX2*	ENST00000603300	0.030	18.373
A_21_P0005252		*LOC340340*	ENST00000470135	0.010	15.816
A_32_P65628	NM_001008387	*REG3G*	ENST00000272324	0.004	12.690
A_33_P3531373	XR_252283	*LOC340340*	ENST00000602761	0.003	9.361
A_23_P2920	NM_001085	*SERPINA3*	ENST00000393078	0.037	7.244
A_24_P92472	NM_000204	*CFI*	ENST00000618244	0.045	7.119
A_21_P0003971	XM_006714503	*lnc-ROPN1L-1*		0.018	6.702
A_23_P137856	NM_002456	*MUC1*		0.008	6.259
A_22_P00009177	CF593671	*LOC102725393*	ENST00000565072	0.015	5.765
A_33_P3285602			ENST00000372451	0.026	5.285
A_21_P0003739		*lnc-AC021860.1-4*		0.040	5.099
A_21_P0012134			ENST00000340424	0.031	4.679
A_22_P00011660	CR746194	*lnc-PDE4D-2*		0.041	4.653
A_33_P3340454				0.002	4.603
A_21_P0012435	NR_110135	*LINC01267*	ENST00000502417	0.038	4.496
A_21_P0012278			ENST00000434304	0.024	4.450
A_21_P0003203		*lnc-FGF12-2*		0.006	4.367
A_22_P00020831			ENST00000502861	0.021	4.367
A_22_P00008249	NR_103859	*LINC01082*	ENST00000601250	0.001	4.273
A_22_P00003732			ENST00000563061	0.017	4.206
A_21_P0011379		*XLOC_l2_004954*		0.033	4.176
A_33_P3419875				0.020	4.121
A_21_P0012181		*XLOC_l2_009135*		0.011	4.114
A_22_P00013293		*LOC157273*	ENST00000522836	0.048	3.970
A_33_P3343705	NR_037597	*LOC442028*	ENST00000582835	0.012	3.928
A_21_P0000009	NM_001145729	*LMNTD1*		0.003	3.920
A_21_P0002140		*lnc-OBSL1-1*		0.043	3.874
A_21_P0014153	NR_125875	*LOC102724201*		0.001	3.873
A_22_P00021841	BX113914	*lnc-NDUFAF2-1*		0.014	3.839
A_21_P0010132	NR_024089	*LINC00162*	ENST00000615826	0.025	3.827
A_21_P0006379		*lnc-B4GALT1-1*		0.007	3.821
A_23_P395464	NM_144690	*ZNF582*	ENST00000619584	0.001	3.816
A_33_P3655815	BQ322426	*SNORA2B*		0.018	3.815
A_23_P64173	NM_001017534	*CARD16*		0.040	3.797
A_21_P0006877		*lnc-REEP3-2*		0.036	3.788
A_21_P0004882		*lnc-UBR2-2*		0.015	3.754
A_22_P00015761			ENST00000584566	0.014	3.749
A_33_P3378684				0.002	3.748
A_22_P00014357	NR_104137	*ARMC2-AS1*		0.039	3.723
A_23_P340848	NM_000960	*PTGIR*	ENST00000291294	0.043	3.700
A_23_P218442	NM_002483	*CEACAM6*	ENST00000199764	0.027	3.679
A_22_P00012150	BC059953		ENST00000564240	0.000	3.650
A_22_P00014429		*lnc-SGSM3-1*		0.048	3.639
A_32_P26401	NR_024102	*LINC01548*	ENST00000451980	0.037	3.634
A_21_P0009569		*lnc-CDH19-1*		0.028	3.589
A_21_P0013696	XM_006726491		ENST00000427989	0.010	3.570
A_21_P0005851	XR_426327	*LOC102724687*		0.045	3.563
A_21_P0004157			ENST00000515153	0.006	3.546
A_22_P00021046			ENST00000567538	0.020	3.541
A_22_P00025465		*lnc-C1orf124-2*		0.030	3.522
A_21_P0008322			ENST00000554829	0.031	3.500
A_32_P10936	NM_004061	*CDH12*	ENST00000382254	0.005	3.489
A_33_P3332215	NM_001044392	*MUC1*		0.028	3.486
A_19_P00319981	NR_120685	*LINC01503*	ENST00000436710	0.037	3.450
A_22_P00021365		*lnc-C9orf156-2*		0.019	3.433
A_23_P14351	NM_004274	*AKAP6*		0.042	3.427
A_22_P00004446	NR_105017	*LOC100505878*	ENST00000503843	0.005	3.413
A_22_P00009915			ENST00000414581	0.003	3.372
A_24_P92174	DQ100868		ENST00000390597	0.018	3.358
A_22_P00000779		*lnc-ADD3-3*		0.022	3.349
A_21_P0007366		*lnc-ORAOV1-4*		0.010	3.348
A_33_P3329922	NR_103829	*CCNYL2*	ENST00000483242	0.049	3.332
A_22_P00000156		*lnc-ABCA5-2*		0.011	3.311
A_22_P00021012		*lnc-GLS-1*		0.035	3.311
A_22_P00006723	DB486346		ENST00000549261	0.029	3.305
A_19_P00315725		*LOC100422737*	ENST00000418134	0.018	3.298
A_21_P0008289	NR_073460	*BMS1P17*	ENST00000444357	0.024	3.289
A_33_P3248948				0.000	3.283
A_22_P00023677	XR_433584		ENST00000556021	0.036	3.268
A_33_P3370019	AK093943		ENST00000390556	0.028	3.267
A_21_P0013336		*XLOC_l2_013921*		0.035	3.258
A_32_P113066	NM_004744	*LRAT*	ENST00000510733	0.013	3.256
A_21_P0005126		*lnc-LIN28B-3*		0.015	3.214
A_24_P58204	NM_001005567	*OR51B5*	ENST00000300773	0.035	3.203
A_23_P385105	NM_032726	*PLCD4*	ENST00000473443	0.016	3.201
A_33_P3304065	NM_000486	*AQP2*	ENST00000199280	0.046	3.197
A_22_P00022879		*lnc-SMAD5-1*		0.028	3.189
A_21_P0001379		*lnc-PCP4L1-1*		0.025	3.188
A_21_P0004764		*lnc-SNAP91-1*		0.022	3.166
A_22_P00017782	NR_103839	*FRY-AS1*		0.028	3.164
A_22_P00006986		*lnc-GDPD4-2*		0.007	3.152
A_33_P3382031	NM_001098844	*TMEM236*	ENST00000377495	0.011	3.145
A_22_P00019546		*lnc-RADIL-1*		0.014	3.119
A_22_P00004133			ENST00000522661	0.044	3.115
A_22_P00009406	NR_104147	*LOC100996447*		0.011	3.108
A_21_P0005426		*lnc-ARF5-5*		0.010	3.108
A_21_P0009531		*lnc-MPPE1-1*		0.006	3.092
A_22_P00018708	XR_241962	*LOC101927913*		0.038	3.089
A_23_P62890	NM_002053	*GBP1*	ENST00000370473	0.007	3.086
A_22_P00003111		*lnc-C9orf103-2*		0.025	3.079
A_23_P50638	NM_052972	*LRG1*	ENST00000306390	0.005	3.066
A_22_P00015445	XR_109653	*LOC100505664*	ENST00000613502	0.001	3.031
A_33_P3362267	NR_026651	*DGCR10*		0.004	3.030
A_24_P228302	NM_006890	*CEACAM7*	ENST00000006724	0.022	3.026
A_21_P0012959			ENST00000513133	0.008	3.016
A_23_P104798	NM_001562	*IL18*	ENST00000528832	0.008	2.977
A_22_P00014513	AK091594	*lnc-SHPRH-2*		0.000	2.966
A_21_P0001033			ENST00000428646	0.017	2.962
A_33_P3344414	NM_001004064	*OR8J3*	ENST00000301529	0.021	2.955
A_21_P0009397	NR_110845	*LOC101928674*	ENST00000587575	0.024	2.949
A_23_P76386	NM_003044	*SLC6A12*		0.022	2.942
A_33_P3330886	NM_001005518	*OR6C65*	ENST00000379665	0.026	2.939
A_23_P356163	NM_178824	*WDR49*	ENST00000308378	0.042	2.937
A_22_P00016627	NR_110812	*TMEM108-AS1*		0.036	2.923
A_22_P00016562	XR_243598	*LOC284191*	ENST00000428142	0.049	2.913
A_23_P153320	NM_000201	*ICAM1*	ENST00000264832	0.014	2.894
A_21_P0003303	NR_109968	*LINC01208*	ENST00000434969	0.000	2.893
A_33_P3267577	NM_003007	*SEMG1*	ENST00000372781	0.037	2.879
A_22_P00014064	AK055411		ENST00000577382	0.025	2.863
A_33_P3295154	AK023831			0.026	2.854
A_21_P0005531		*lnc-AKR1B1-1*		0.023	2.850
A_23_P16275	NM_021733	*TSKS*	ENST00000358830	0.017	2.838

Probe NAME	GB_ACC	GENE_SYMBOL	ENSEMBL_ID	P-Value	Fold Change
A_19_P00805339		*LOC149351*	ENST00000455680	0.040	2.832
A_33_P3321564				0.011	2.822
A_21_P0003662		*lnc-LSM6-1*		0.031	2.813
A_33_P3358774	NM_001005212	*OR9Q1*	ENST00000612174	0.049	2.809
A_22_P00006117			ENST00000512284	0.037	2.792
A_21_P0001492	NR_038928	*LINC01343*	ENST00000431311	0.010	2.785
A_21_P0010242		*lnc-DYRK1A-1*		0.006	2.772
A_23_P126363	NM_021794	*ADAM30*	ENST00000369400	0.001	2.769
A_23_P151975	NM_016321	*RHCG*	ENST00000558360	0.014	2.767
A_23_P47967	NM_022363	*LHX5*		0.039	2.766
A_22_P00004540	AK123984	*lnc-CREG1-3*		0.006	2.757
A_22_P00017402		*lnc-VCAN-1*		0.004	2.752
A_33_P3294583	AK123926	*LOC256880*		0.044	2.752
A_21_P0004876		*lnc-AL353597.1-2*		0.022	2.749
A_21_P0009718		*lnc-HAUS5-2*		0.018	2.744
A_21_P0002251		*lnc-ATOH8-2*		0.029	2.736
A_33_P3236109	NM_000275	*OCA2*	ENST00000354638	0.018	2.725
A_24_P91830	NM_001009609	*SPANXN3*	ENST00000370503	0.019	2.715
A_23_P116414	NM_007069	*PLA2G16*	ENST00000394613	0.002	2.698
A_21_P0009675		*lnc-PTBP1-1*		0.005	2.673
A_22_P00011542			ENST00000505436	0.003	2.672
A_33_P3802966				0.027	2.669
A_33_P3312774				0.010	2.662
A_24_P139665	NM_016257	*HPCAL4*	ENST00000372844	0.011	2.651
A_33_P3280360	AK128800	*LOC100133857*		0.009	2.649
A_22_P00010058		*lnc-MON2-3*		0.038	2.648
A_32_P159535			ENST00000445502	0.046	2.639
A_33_P3276604			ENST00000598428	0.040	2.636
A_22_P00012092	NR_103559	*CDRT8*	ENST00000579752	0.040	2.633
A_33_P3241844	AK126625	*ZNF551*		0.009	2.633
A_21_P0014555	XR_110289	*LOC100506446*	ENST00000556434	0.033	2.623
A_21_P0008822	XR_429515	*LOC102724283*		0.017	2.619
A_21_P0000991			ENST00000419658	0.048	2.612
A_24_P710999			ENST00000441416	0.033	2.604
A_33_P3368034				0.032	2.598
A_22_P00017098	NR_049724	*GRM5-AS1*		0.016	2.588
A_23_P26583	NM_032206	*NLRC5*	ENST00000545081	0.024	2.586
A_21_P0012587		*XLOC_l2_010691*		0.044	2.579
A_33_P3231065				0.040	2.577
A_21_P0012639		*XLOC_l2_010963*		0.015	2.571
A_19_P00808794		*XLOC_l2_008221*		0.042	2.566
A_22_P00004262			ENST00000425125	0.036	2.560
A_21_P0005468		*lnc-RNF216-1*		0.026	2.553
A_21_P0000564				0.032	2.553
A_33_P3217639	NM_001099667	*ARMS2*	ENST00000528446	0.028	2.532
A_22_P00018279			ENST00000414548	0.039	2.530
A_33_P3339611				0.021	2.529
A_22_P00000833		*lnc-AGAP10-3*		0.024	2.528
A_21_P0014408	NR_040513	*LOC100506384*		0.027	2.526
A_21_P0007495		*lnc-BEST3-1*		0.004	2.525
A_33_P3342175	AK126607			0.044	2.524
A_22_P00010648	AA453185		ENST00000522570	0.008	2.515
A_23_P375372	NM_021871	*FGA*	ENST00000403106	0.041	2.501
A_21_P0014689	XR_111668	*LOC100506974*		0.037	2.495
A_33_P3221916				0.040	2.492
A_21_P0003736		*lnc-ARAP2-7*		0.001	2.487
A_33_P3384902	NM_001005188	*OR6X1*	ENST00000327930	0.023	2.460
A_22_P00003053			ENST00000419596	0.034	2.452
A_21_P0012714	NR_033931	*LINC01085*	ENST00000511200	0.030	2.449
A_21_P0010625		*XLOC_l2_000945*		0.033	2.441
A_23_P257803	NM_013391	*DMGDH*	ENST00000255189	0.042	2.437
A_21_P0010033		*lnc-PLAGL2-2*		0.017	2.436
A_21_P0010337		*lnc-RABL2B-2*		0.005	2.435
A_21_P0010201		*lnc-TMEM50B-1*		0.031	2.420
A_24_P188377	NM_000574	*CD55*		0.050	2.416
A_21_P0003661		*lnc-LSM6-1*		0.047	2.414
A_24_P118052	NM_199254	*TPTE2*		0.016	2.405
A_33_P3354579				0.020	2.398
A_21_P0000906	NR_040250	*WWTR1-AS1*		0.020	2.382
A_22_P00006194		*lnc-FAM26E-1*		0.012	2.379
A_21_P0006398		*lnc-BRD3-2*		0.014	2.379
A_22_P00015437	NR_046617	*KCNAB1-AS2*		0.012	2.375
A_21_P0013152			ENST00000430296	0.027	2.372
A_33_P3405424	NM_152899	*IL4I1*		0.036	2.361
A_21_P0003696		*lnc-ZFP42-2*		0.036	2.361
A_22_P00019088		*lnc-SLC45A1-1*		0.038	2.345
A_21_P0014737	BG191046	*ZFPM2*	ENST00000521923	0.035	2.330
A_22_P00004299		*lnc-CNR1-1*		0.039	2.327
A_21_P0005000		*lnc-CCDC90A-1*		0.022	2.326
A_21_P0014193				0.043	2.324
A_22_P00004074		*lnc-CIB2-1*		0.014	2.318
A_32_P332551	NM_182508	*FAM216B*	ENST00000537894	0.022	2.313
A_22_P00015854	BX487629		ENST00000428516	0.035	2.310
A_33_P3365200				0.009	2.309
A_32_P928190	NR_046245	*LOC100130964*	ENST00000519537	0.022	2.303
A_21_P0007090		*lnc-PITRM1-7*		0.050	2.285
A_33_P3855484				0.050	2.275
A_22_P00019707	DA593750		ENST00000523232	0.036	2.274
A_33_P3325653			ENST00000473520	0.043	2.270
A_22_P00001770		*lnc-ATP1A1OS-1*		0.035	2.256
A_22_P00008133	XR_246588	*LOC101927783*		0.023	2.255
A_21_P0014182	AK094662	*LOC101927354*		0.045	2.251
A_33_P3214670	NM_000189	*HK2*	ENST00000290573	0.042	2.244
A_21_P0002153		*lnc-ACP1-6*		0.016	2.234
A_33_P3411975	NM_015429	*ABI3BP*	ENST00000475896	0.048	2.227
A_23_P355980	AB002058	*P2RX6*	ENST00000413302	0.003	2.219
A_21_P0000706	NR_037903	*BHLHE40-AS1*		0.022	2.218
A_22_P00019068		*lnc-CLEC14A-2*		0.050	2.217
A_23_P87007	NM_006028	*HTR3B*	ENST00000537778	0.037	2.216
A_22_P00001935			ENST00000563190	0.036	2.212
A_21_P0007424		*lnc-SPATA19-1*		0.035	2.202
A_23_P49559	NM_181790	*GPR142*	ENST00000335666	0.029	2.196
A_33_P3236516			ENST00000419302	0.041	2.172
A_33_P3387493	NM_001271682	*FTH1P18*		0.018	2.168
A_21_P0006532			ENST00000602507	0.009	2.144
A_21_P0008117		*lnc-COL4A2-2*		0.024	2.137
A_24_P923945	AK022322	*UBR4*	ENST00000375218	0.043	2.134
A_23_P140074	NM_003891	*PROZ*	ENST00000375547	0.038	2.125
A_23_P121413	NM_153330	*DNAJB8*	ENST00000319153	0.043	2.122
A_23_P139786	NM_003733	*OASL*	ENST00000257570	0.034	2.120
A_22_P00005480	BX091138	*lnc-DYNLRB2-1*		0.039	2.119
A_33_P3238773				0.017	2.098
A_33_P3409765	XM_006716168	*MGAM*		0.042	2.088
A_23_P207879	NM_024110	*CARD14*	ENST00000575500	0.005	2.083
A_22_P00004476	AK094838	*lnc-CPM-1*		0.035	2.080
A_19_P00321190			ENST00000521132	0.035	2.070
A_23_P131089	NM_198471	*KANK3*	ENST00000330915	0.019	2.047
A_22_P00000015	AK054921	*lnc-RP1-177G6.2.1-2*		0.026	2.037
A_24_P41850	NM_001031849	*MASP1*	ENST00000169293	0.047	2.018

Supplementary Table S4

Downregulated genes in CRC organoids with co-culture with CAFs.

Microarray probes with *P*<0.05, Fold change<0.5, are shown.

Probe NAME	GB_ACC	GENE_SYMBOL	ENSEMBL_ID	P-Value	Fold Change
A_22_P00003407			ENST00000515199	0.023	0.193
A_33_P3333648	NM_001004754	*OR51I2*	ENST00000341449	0.028	0.216
A_22_P00020724	NR_120652	*LINC01517*	ENST00000426922	0.036	0.268
A_23_P256735	NM_016134	*CPQ*	ENST00000220763	0.044	0.296
A_23_P65240	NM_001845	*COL4A1*	ENST00000375820	0.045	0.311
A_21_P0012909		*XLOC_l2_012071*		0.017	0.316
A_24_P195081	NM_173550	*CCDC171*		0.034	0.324
A_21_P0014727	NR_040671	*HMGN3-AS1*		0.003	0.327
A_33_P3358893	NM_001286359	*PRM2*		0.004	0.331
A_23_P211973	NM_024800	*NEK11*	ENST00000510474	0.027	0.332
A_23_P137157	NM_002910	*RENBP*	ENST00000442361	0.037	0.335
A_22_P00018473		*lnc-TSSC4-1*		0.002	0.341
A_33_P3336273	NM_001024383	*NAV3*	ENST00000397909	0.008	0.350
A_33_P3329104				0.003	0.352
A_22_P00002022	NR_125866	*LOC101930010*		0.001	0.355
A_23_P151895	NM_003613	*CILP*		0.024	0.355
A_22_P00003373	BM711518		ENST00000570408	0.046	0.355
A_21_P0005062		*lnc-TXLNB-2*		0.023	0.358
A_22_P00012316		*lnc-PPP6R2-1*		0.006	0.364
A_32_P179998	NM_033053	*DMRTC1*	ENST00000334036	0.001	0.365
A_21_P0014194				0.024	0.370
A_22_P00009140	XR_248480	*LOC101928144*	ENST00000582983	0.016	0.374
A_32_P183918	NR_033851	*SERPINB9P1*	ENST00000420981	0.030	0.377
A_21_P0004623		*lnc-AL035696.1-3*		0.032	0.382
A_23_P139198	NM_033101	*LGALS12*		0.001	0.387
A_22_P00010256		*lnc-MTERFD2-2*		0.017	0.390
A_21_P0001765	NR_033875	*LINC00954*	ENST00000449086	0.024	0.393
A_21_P0007500		*lnc-CD9-1*		0.012	0.393
A_33_P3333267	NM_001204210	*TMEM235*	ENST00000551068	0.004	0.404
A_23_P102706	NM_014723	*SNPH*	ENST00000614659	0.009	0.405
A_33_P3342720	NM_173201	*ATP2A1*	ENST00000357084	0.031	0.406
A_21_P0000390	NR_003074	*SNORD92*		0.026	0.406
A_22_P00015973			ENST00000566922	0.023	0.410
A_33_P3262685	NR_024591	*POM121L1P*		0.020	0.412
A_33_P3350575	NM_001080399	*OC90*	ENST00000254627	0.028	0.413
A_22_P00000382	NR_126005	*KRT73-AS1*		0.035	0.414
A_21_P0010137	NR_033840	*LINC00163*	ENST00000434081	0.030	0.415
A_23_P99996	NM_019066	*MAGEL2*	ENST00000532292	0.046	0.417
A_22_P00025615	DB031156		ENST00000582755	0.020	0.417
A_23_P156788	NM_003764	*STX11*	ENST00000367568	0.018	0.418
A_33_P3391511				0.015	0.425
A_21_P0006510		*lnc-PRPS1-1*		0.043	0.425
A_22_P00007341	NR_038920	*LOC100506314*		0.036	0.428
A_33_P3280147	NR_028337	*FLJ45079*	ENST00000374983	0.024	0.428
A_22_P00010321			ENST00000451108	0.002	0.429
A_22_P00009258	XR_249026		ENST00000432495	0.009	0.430
A_33_P3423171	NM_018031	*WDR6*	ENST00000488572	0.049	0.432
A_23_P259292	NM_015645	*C1QTNF5*	ENST00000530681	0.030	0.432
A_22_P00006989			ENST00000557350	0.008	0.434
A_33_P3270369	AK130406			0.030	0.434
A_21_P0004896		*lnc-BAI3-2*		0.037	0.435
A_19_P00324359		*lnc-MYC-1*		0.013	0.438
A_33_P3361758	NM_152647	*FAM227B*	ENST00000299338	0.029	0.441
A_22_P00007215	BQ435778	*lnc-GPC6-2*		0.005	0.445
A_22_P00003873	AK026947	*lnc-CEMP1-5*		0.015	0.445
A_23_P142125	NM_002152	*HRC*	ENST00000252825	0.001	0.450
A_24_P202329	NR_026899	*LOC146880*	ENST00000578036	0.017	0.451
A_22_P00000504		*lnc-AC105020.1-1*		0.001	0.451
A_24_P9833	NM_001256696	*PRDM11*	ENST00000530656	0.021	0.454
A_23_P206022	NM_001004439	*ITGA11*	ENST00000315757	0.005	0.455
A_33_P3245415	NM_001286459	*N4BP2L1*	ENST00000613078	0.011	0.456
A_19_P00316622	XR_424934	*LINC00908*	ENST00000583578	0.005	0.457
A_21_P0011521		*XLOC_l2_005692*		0.028	0.457
A_23_P20578	NM_001164310	*FAM166B*		0.040	0.458
A_22_P00002569		*lnc-C17orf89-2*		0.020	0.463
A_22_P00020522	NR_046834	*RNF216-IT1*		0.004	0.466
A_33_P3360077	AK128206		ENST00000609755	0.033	0.466
A_22_P00013894	NR_044997	*HCG25*		0.026	0.467
A_22_P00006326	BG218223	*lnc-FAM9C-2*		0.003	0.469
A_21_P0001449		*lnc-SCCPDH-1*		0.028	0.474
A_33_P3336657	NM_001080475	*PLEKHM3*	ENST00000427836	0.019	0.475
A_22_P00009210		*lnc-LPIN2-1*		0.029	0.476
A_21_P0001300		*lnc-BSND-1*		0.036	0.476
A_22_P00003095	NR_125827	*LOC102724710*	ENST00000523284	0.016	0.478
A_23_P432005	NM_152670	*TEX37*	ENST00000303254	0.041	0.479
A_21_P0000925	AK022825	*lnc-SCCPDH-1*		0.004	0.479
A_33_P3365755	NM_001136504	*SYT2*	ENST00000367267	0.003	0.479
A_23_P44663	NM_006220	*SERPINA2*	ENST00000553483	0.036	0.481
A_22_P00004724	NR_046217	*ZRANB2-AS2*	ENST00000600103	0.018	0.484
A_22_P00023909	NR_110649	*LOC100507534*	ENST00000570024	0.045	0.485
A_33_P3416797	AL831947	*OVOS2*		0.009	0.488
A_21_P0005293			ENST00000440034	0.029	0.488
A_22_P00004149	NR_104657	*LOC101928035*	ENST00000562888	0.015	0.489
A_21_P0013651		*XLOC_l2_015213*		0.016	0.490
A_22_P00007990		*lnc-IFFO1-1*		0.025	0.490
A_21_P0013503	XM_006725099	*LOC101928108*		0.043	0.492
A_21_P0001397		*lnc-TSEN15-1*		0.033	0.492

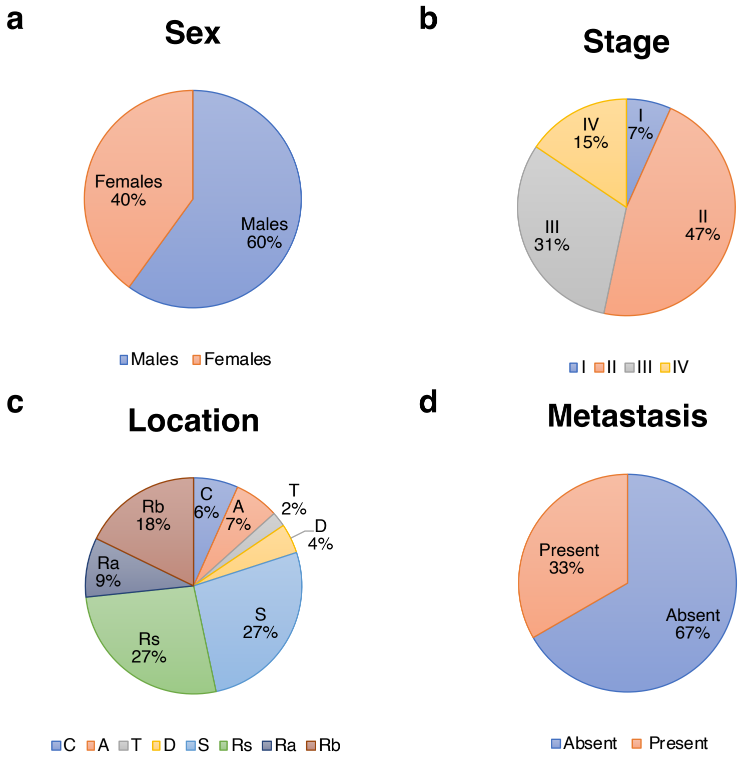

Supplementary Figure. S1

Summary of principle basic and pathological characteristics of 45 CRC patients. (a) Sex ratio of CRC patients. (b) Location of original CRC. (c) The pathological stage of each CRC case. (d) The presence of metastases.

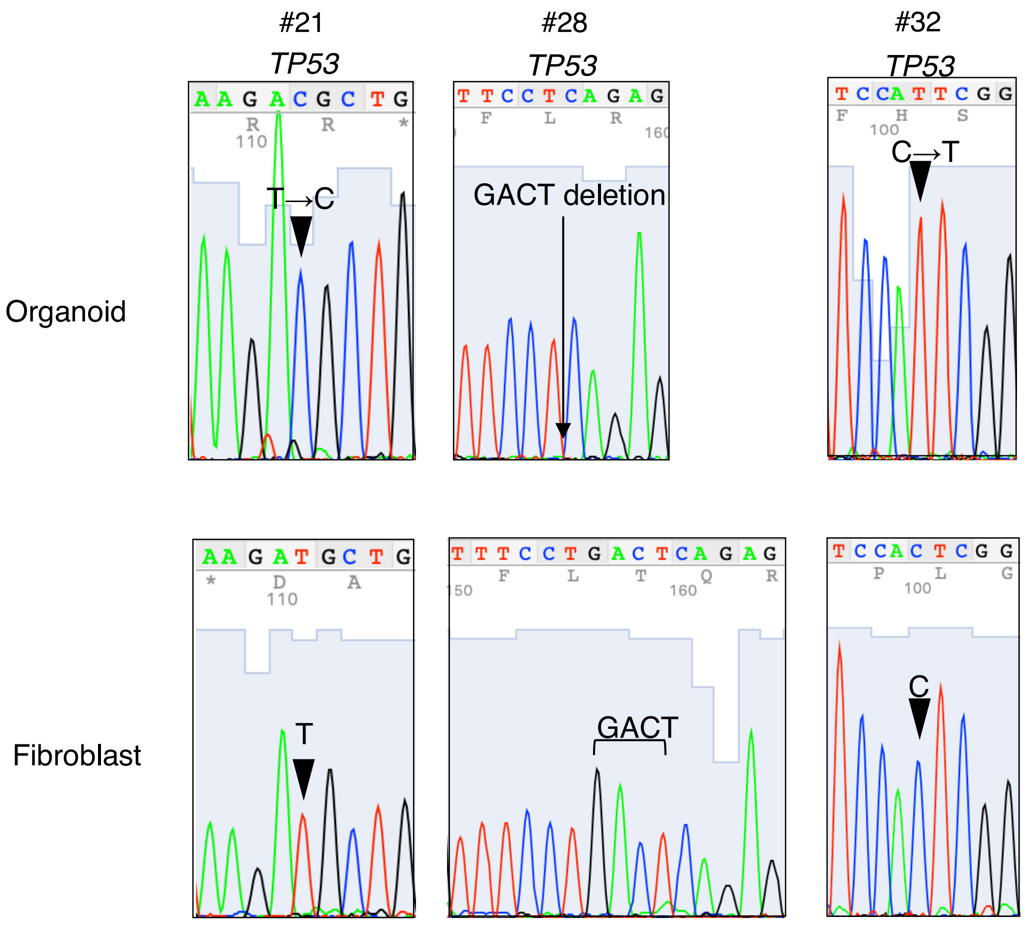

Supplementary Fig. S2

Confirmation of *TP53* mutation/deletion by direct sequencing in organoids, but not in fibroblasts. Cancer mutations in *TP53* for #21, #28, and #32 CRC organoids (upper panels) were absent in fibroblasts.

a

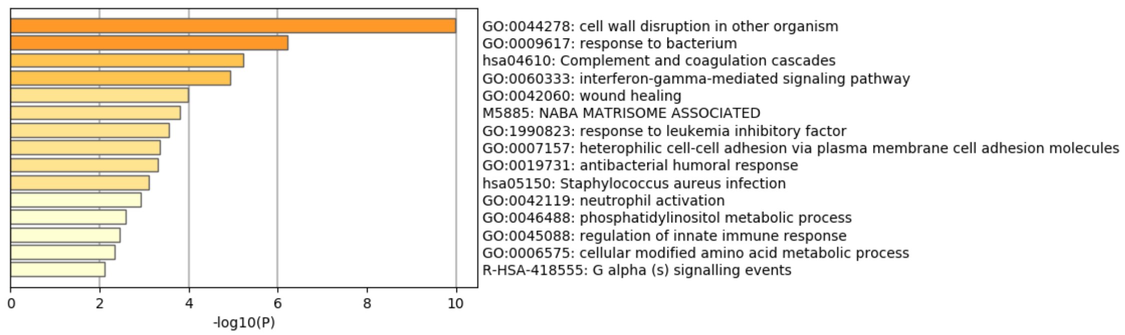

Supplementary Fig. S3

Gene Ontology (GO) term enrichment status for CAF-induced genes. a) CAF-induced genes upregulated by 2-fold (p<= 0.05) were extracted from microarray data for CRC organoids with and without CAFs. The graph displays term enrichment levels along with the GO term hierarchy within the "biological process" branch, and the analysis was performed using Metascape.

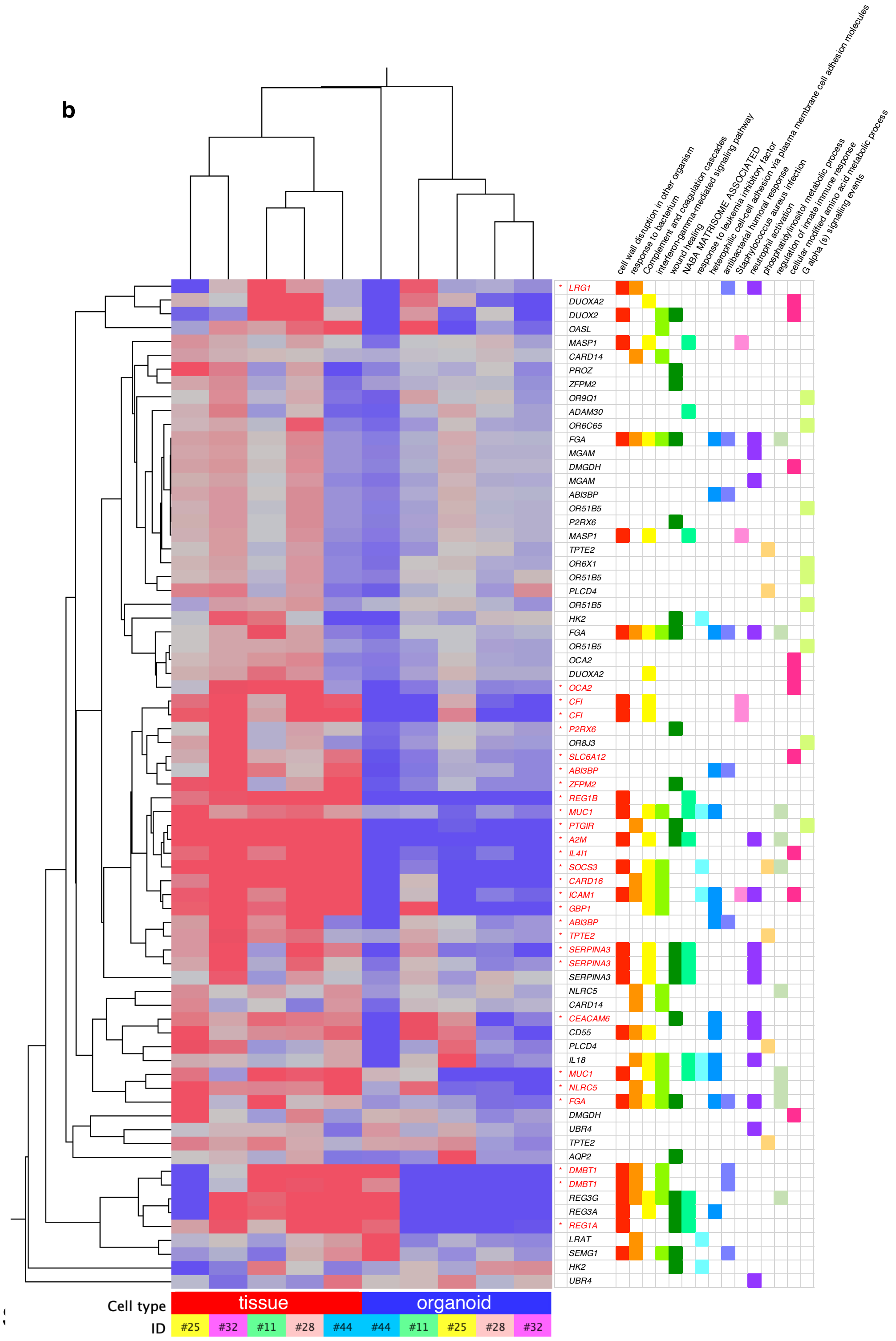

b) Tree clustering of GO term-enriched genes in CRC tissues and CRC organoids without CAFs is shown on the left side of the panel. The enriched GO terms are shown on the right side of the panel. Red-colored genes show a significant ($p \leq 0.05$) 2-fold or greater change in gene expression between tissue and organoid.
